# Transcriptomic Insights into Metabolic Reprogramming and Exopolysaccharide Synthesis in *Porphyridium purpureum* Under Gradual Nitrogen Deprivation

**DOI:** 10.3390/md24010040

**Published:** 2026-01-13

**Authors:** Maurean Guerreiro, Coline Emmanuel, Céline Dupuits, Christine Gardarin, Said Mouzeyar, João Varela, Jane Roche, Céline Laroche

**Affiliations:** 1 UMR CNRS 6602 Institut Pascal, Clermont Auvergne INP, Université Clermont Auvergne, 63000 Clermont-Ferrand, France; guerreiro.maurean@outlook.com (M.G.);; 2 Centre of Marine Sciences, University of Algarve, 8005-139 Faro, Portugal; 3 UMR 1095 Génétique, Diversité et Ecophysiologie des Céréales, Université Clermont Auvergne, INRAE, 63000 Clermont-Ferrand, Francejane.roche@uca.fr (J.R.)

**Keywords:** *Porphyridium*, polysaccharides, nitrogen, deprivation, transcriptomics

## Abstract

*Porphyridium* species are known red microalgae for producing valuable bioactive compounds such as sulfated exopolysaccharides (EPS) with diverse industrial biomedical applications due to their functional and rheological properties. Recent studies have investigated how abiotic stresses, particularly nitrogen deprivation, affect *Porphyridium*’s metabolic regulation and EPS production through transcriptomic analysis. Still, the mechanisms governing EPS biosynthesis and the involvement of carbohydrate-activated enzymes (CAZymes) remain poorly understood. This study investigated the progressive effects of nitrate consumption on the unicellular red alga, *P. purpureum*, by integrating physiological, biochemical, and transcriptomic analyses through RNA-Seq, further validated by RT-qPCR. *P. purpureum* displayed a gradual, phase-dependent metabolic response to progressive nitrogen stress. EPS release coincided with the decline in nitrate uptake, linking nitrogen availability to carbon redirection towards polysaccharide secretion. Transcriptomic data revealed global metabolic downregulation with targeted upregulation of stress-responsive, carbohydrate catabolic, and nucleotide–sugar synthesis pathways, including the upregulation of CAZyme families GT4, GT8, and GT77. Our results give insights into the coordinated nitrogen and carbon metabolic regulation underlying polysaccharide biosynthesis, while opening future perspectives on enzyme compartmentalization and regulatory flux distribution under nitrogen stress in *P. purpureum*.

## 1. Introduction

Rhodophyta represents one of the most ancient lineages of photosynthetic eukaryotes with distinctive plastid and genome features [1,2]. Beyond their phylogenetic importance, many marine seaweed and microalgae species are commercially valuable sources of high-value biocompounds for diverse industrial applications [3,4,5]. Among red microalgae, *Porphyridium* species are highly recognized for their capacity to produce multiple bioactive compounds such as sulfated extracellular polysaccharides (EPSs), phycobiliproteins, and polyunsaturated fatty acids (PUFASs) [6]. Microalgal EPSs, specially from *Porphyridium* spp., are known as interesting alternatives for natural polymer applications due to their functional substituents such as methyl and sulfate groups [7]. Their distinctive monosaccharide composition and high degree of sulfation have been associated with bioactive properties including antitumoral [8], antimicrobial [9], and antiviral effects [10,11,12]. The additional ease of recovery of these natural polymers directly in the media and their desirable rheological properties [13,14] justify the industrial production of EPS for diverse applications including plant biostimulants [15], food dyes, animal feed, and skincare products [3].

Over the past decade, a growing number of studies have been conducted on the effect of abiotic stress, particularly nitrogen deprivation, on the yield of single and/or co-production of high-value compounds in *Porphyridium* species [13,16,17,18,19]. Nitrogen is elemental to photosynthetic organisms, whether for the synthesis of photosynthetic pigments [20] and overall metabolic homeostasis [21]. While physiological data reveal the outcomes of nitrogen deficiency on metabolism and polysaccharide accumulation, transcriptomic analysis elucidates the underlying gene expression variations at the heart of the metabolic reprogramming [22,23].

To our knowledge, *Porphyridium purpureum* is the only mesophilic photosynthetic red microalga among sequenced Rhodophyta genomes [24], offering insight into red algal biology beyond extremophilic contexts [25]. Its fully sequenced genome, since revisited [26,27], and small intron-poor genome size (<20 Mb) facilitate advanced molecular studies, including nuclear, plastid genetic transformation, plasmid replication, and CRISPR-Cas9 gene editing [24,28,29]. The combination between genetic availability and industrial interest makes *P. purpureum* a robust model for studying metabolic regulation at the transcriptome level. Recent transcriptomic studies have substantially advanced the knowledge of nitrogen stress responses in *P. purpureum*. Clear insights into the immediate metabolic adaptation of *P. purpureum* under nitrogen stress were provided by [22], demonstrating that carbon flow is rapidly redirected toward polysaccharide synthesis as a first response, followed by lipid synthesis during prolonged nitrogen deprivation. Similar transcriptomic reprogramming in *P. cruentum* was reported by [23], including the upregulation of nitrate assimilation, carbon/nitrogen metabolism shifts, downregulation of photosynthesis genes, and modifications in carbohydrate, lipid, and protein metabolism. Building on this, [30] provided detailed insights of lipid metabolism in *P. cruentum* under nitrogen limitation through lipidomics and transcriptomics, particularly of long-chain (LC-) PUFA synthesis pathways and glycerolipid metabolism. Indeed, the literature offers multiple transcriptomics studies elucidating lipid accumulation under nitrogen stress, particularly in oleaginous microalgae [31,32]. However, EPS-producing microalgae and, particularly, the regulation of biosynthetic enzymes, such as carbohydrate-active enzymes (CAZymes), under nitrogen stress remains poorly understood [10]. Questions persist regarding the initial steps of polysaccharide synthesis, their polymerization, structural modification, and their release in the media [22].

Our study addresses these questions by focusing on the natural progression of nitrate consumption in *P. purpureum* instead of an abrupt nitrogen deprivation shift. By monitoring physiological changes, EPS production, final EPS composition, and transcriptomic dynamics throughout the cultivation process, we aimed to uncover the nuanced metabolic transitions and gain new insights on polysaccharide synthesis. We also aimed towards giving further insights regarding the regulation of CAZymes under nitrogen stress and their role in polysaccharide metabolism. Transcriptome-wide pairwise comparisons were conducted across nitrogen-replete, nitrogen-limited, and late-stage nitrogen-depleted conditions using differential gene expression, Gene Ontology (GO), and KEGG pathway enrichment analyses. A subset of candidate CAZyme genes were validated through RT-qPCR to confirm their transcriptional responses to nitrogen fluctuations.

## 2. Results and Discussion

### 2.1. Significant Exopolysaccharide (EPS) Release Coincides with Nitrate Uptake Rate Decline, Hinting at Porphyridium purpureum Shift into Nitrogen-Limiting Conditions

**Figure 1:**
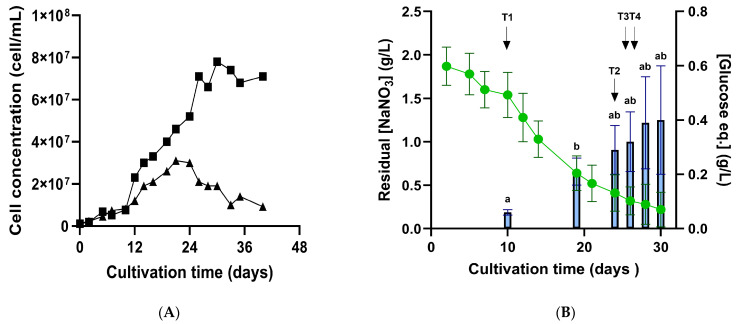
Growth (**A**) nitrate consumption and exopolysaccharide (EPS) production (**B**) during 5L-photobioreactors cultivation of *P. purpureum*. (**A**) Cell concentration (cells/mL) shown as individual growth curves from two independent biological replicates. (**B**) Residual nitrate concentration (dotted line, g/L) and EPS concentration quantified by total carbohydrate assay and expressed as glucose equivalents (bars, g/L). Data shown as means ± standard deviations (SD) of biological triplicates until day 28 (start of stationary phase). Measurements beyond this timepoint are provided in Dataset S1. Statistically significant differences between timepoints are indicated by distinct letters (*p*-value < 0.05; Student’s *t*-test). RNA-Seq sampling points (T1–T4) are indicated by arrows.

Cell concentration of *Porphyridium purpureum* was monitored over a 30–40-day cultivation period (Figure 1), with sampling taken every 2 to 3 days to assess physiological characterization.

Figure 1A shows the typical growth kinetics of *P. purpureum*, with a lag phase (0–10 days), an exponential phase (14–24 days), and a stationary phase (from day 24 onward). The lag phase reflects microalgal adaptation to new media, while the plateau phase reflects stationary phase entry, resulting from nutrient limitation known to induce high-value compound production, which, in this case, is exopolysaccharide (EPS) synthesis [5,33]. Nitrate uptake was monitored by measuring the residual nitrate concentration and EPS content expressed as glucose equivalents in the medium (Figure 1B). The maximal nitrate uptake rate was 0.14 ± 0.01 g/L per day (Dataset S1). This high rate, higher than that reported by [7], can be attributed to the use of tailored media, designed to target 3 g/L of biomass using a N/P ratio of ~4. This media formulation used here was stoichiometrically designed by [34] for *Porphyridium marinum* and adapted to *P. purpureum* to optimize EPS production through nitrogen stress. EPS release quantification showed a statistically significant increase between day 10 and day 19 (*p* < 0.05, Student *t*-test). This significant EPS release coincided with the onset of nitrogen limitation, demonstrated by a drop in nitrate concentrations of ~40% on day 19 (Dataset S1). Although higher EPS concentrations were expected using this tailored media ([34]: at least 4-fold more in *P. marinum* at 28 °C), our results still align with the literature, estimating maximum EPS concentrations of 0.5–1 g/L for red microalgae [5,35].

### 2.2. P. purpureum EPS Exhibits Consistent Monosaccharide Composition

**Figure 2:**
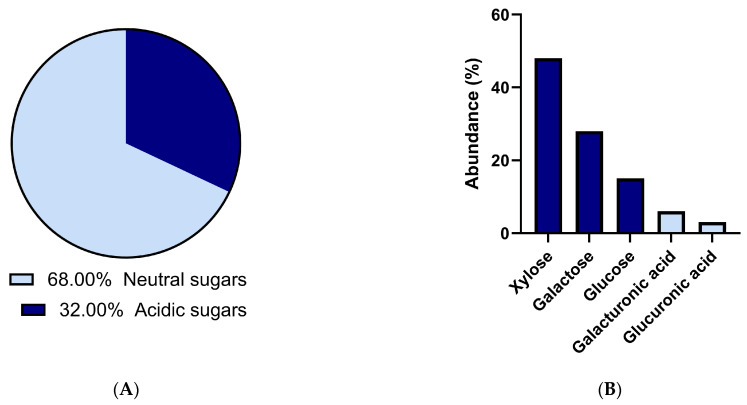
Exopolysaccharide general (**A**) and monosaccharide (**B**) composition. (**A**) Acidic and neutral sugar contents of EPS extracted from one of the biological replicates were expressed in % of mass ratios. (**B**) Monosaccharide composition of EPS determined by HPAEC-PAD from the same biological replicate and are expressed in % of molar ratios (Dataset S2). These data are presented as descriptive compositional reference information and were not subjected to statistical analysis.

Residual culture medium collected at the end of cultivation was desalinated and lyophilized to allow further EPS characterization (Figure 2). Due to the limited availability of EPS material, monosaccharide composition analyses were performed on a single biological replicate and are therefore interpreted descriptively rather than quantitatively.

EPS general sugar content analysis showed a composition of 60% neutral sugars, and the remaining 40% consisted of acidic sugars (Figure 2A). HPAEC-PAD-based analysis of EPS from a single biological replicate revealed monosaccharide composition, with xylose (close to 50% molar) being the dominant neutral monosaccharide, followed by galactose, glucose, and acidic sugars like galacturonic acid and glucuronic acid (Figure 2B). Although no statistical inference can be drawn from this single-replicate dataset, the relative molar proportions of neutral sugars are consistent with [13], reporting 46%, 30%, 20%, and 3.8%, respectively. This composition is consistent with the literature for *Porphyridium purpureum* [10,36,37] and Porphyridiophyceae species, in general [38]. Low-fucose content was consistent with their known abundance in brown algae [22]. Discrepancies between biochemical methods and chromatographic quantifications are likely due to unassigned peaks related to methylated or acetylated uronic acids. This is a key challenge in characterizing *P. purpureum*’s EPS, a point highlighted recently by [13], who were the first to detect methylation in *P. purpureum* EPS. Taken together, while monosaccharide proportions may vary quantitatively with nitrogen availability [16], EPS composition consistently included xylose, glucose, and galactose, confirming a conserved structural backbone characteristic of *P. purpureum* polysaccharides.

While this temporal association is consistent with previous reports linking nitrogen limitation to EPS synthesis [33,39], it remains correlative, and causality cannot be inferred from these data alone. Alternative explanations for increased EPS production could include the transition into the stationary phase, carbon overflow metabolism, or other stress responses induced by culture conditions [7,34,35]. Nonetheless, the observed temporal pattern provides a strong incentive to go beyond physiological characterization and explore transcriptional responses, encouraged by the hypothesis of a potentially conserved biosynthetic machinery underlying the early steps of polysaccharide synthesis.

To give transcriptional insights into polysaccharide synthesis in response to nitrogen stress, four timepoints (T1–T4) were selected, corresponding to distinct stages of *P. purpureum* growth and different nitrogen availabilities. T1, taken at the start of the logarithmic phase, was associated with the nitrogen-replete (NR) condition ([NaNO_3_] = 1.5 g/L); T2 and T3, sampled during the pre-stationary phase, were associated with progressive nitrogen-limitation (NL) conditions ([NaNO_3_] = 0.6 g/L, “NL1”; [NaNO_3_] = 0.5 g/L, “NL2”); and one last sampling at the stationary phase, corresponded to late-stage nitrogen depletion (LND) ([NaNO_3_] = 0.3 g/L), as it was harvested after significant increase in EPS release (Figure 1B). Twelve samples (three technical replicates per timepoint) of RNA extracts were sent to the Genotoul platform to carry out RNA-Seq analysis.

### 2.3. Two-Dimensional-PCA Distinguishes Distinct Transcriptomic Clusters Along Nitrogen Depletion Phases

**Figure 3:**
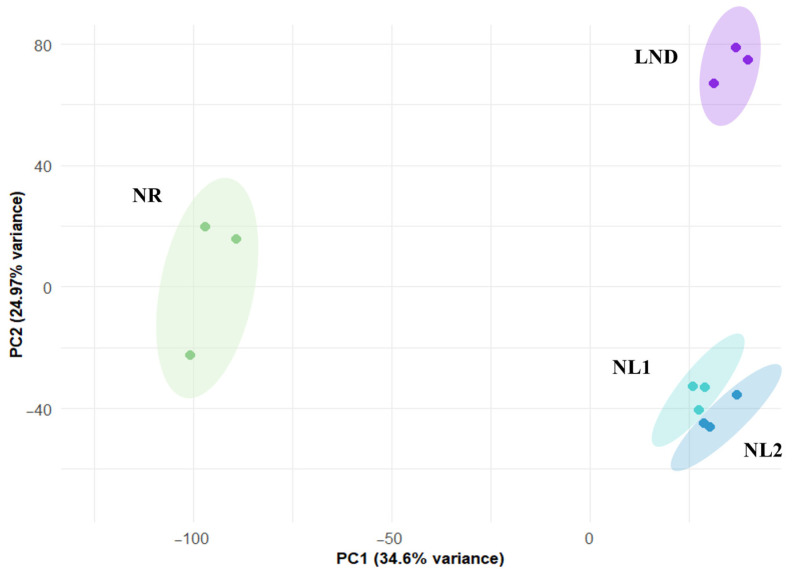
Two-dimensional principal component analysis (2D-PCA) of gene expression taken under nitrogen-replete (NR), nitrogen-limited (NL1, NL2), and late-stage nitrogen-depletion (LND) conditions. Gene expression variance of samples taken at T1, T2-T3, and T4 in technical triplicates.

PCA of normalized gene expression revealed three distinct clusters corresponding to samples T1, T2–T3, and T4 (Figure 3; Dataset S3).

These samples were assigned to nitrogen-replete (NR, green), nitrogen-limited (NL1, turquoise; NL2, blue), and late-stage nitrogen-depleted (LND, purple) conditions based on cell growth, residual nitrates, and total sugar levels. Our results align with [32], who classified the *Phaeodactylum tricornutum* transcriptome under nitrogen stress into an NR group, a transition phase, and a nitrogen-starved group. The proximity of clusters NL1 and NL2 reflects the relatively short time interval between samplings (2 days). Accordingly, intermediate samples T2-T3 were assigned to the “nitrogen-limiting” phase and analyzed separately against both NR and LND conditions. Indeed, this transient phase has been characterized as holding the metabolic shifts from external nitrate availability to internal nitrogen depletion [32], making it a key window for investigating nitrogen/carbon metabolism leading to polysaccharide biosynthesis. Gene annotation of the recovered transcriptome based on the literature reference (contigs from [27]) resulted in the successful identification of 9898 genes (Dataset S3).

### 2.4. Differential Expression Analysis Reveals Gradual Metabolic Mobilization Under Nitrogen Stress

Differentially expressed genes (DEGs) were identified through pairwise comparisons (Table 1; Dataset S4).

**Figure 4:**
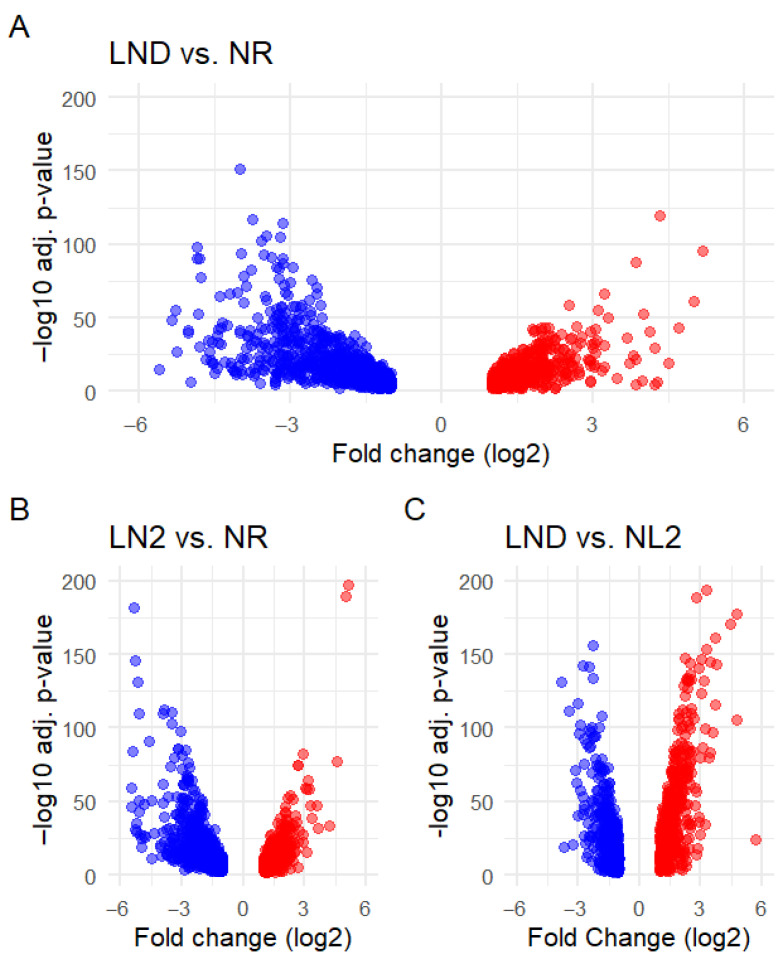
Volcano plots of DEGs across pairwise comparisons: (**A**) LND vs. NR, (**B**) LND vs. NR, and (**C**) NL2 vs. LND. Down (blue)- and upregulated DEGs (red) are plotted according to their fold change (FC, log_2_) and their statistical significance (FDR, –log_10_(adj. *p*-value)). FC threshold was set at |log_2_(FC)| ≥ 1 and FDR threshold was set at –log_10_(adj.pv) ≥ 2. Differential expression analysis was performed using DESeq2 on raw counts from all biological replicates, applying the likelihood ratio test (LRT) to identify DEGs. Volcano plots were generated from the resulting DESeq2 output (Figure S1).

Of the 9898 identified genes, 2769 were annotated as uniquely DEGs (Table S1), indicating that ~28% of gene products from the recovered transcriptome could have been affected by nitrate availability. Among these genes, our study revealed a predominant transcriptional downregulation under NL and LND compared to NR conditions (Table 1A). Inverted patterns were seen in [23] when studying *P. cruentum* under strict LND against NR conditions. DEG numbers increased progressively with decreasing nitrogen availability, peaking during the transition from NR to LND (1687 DEGs). LND to NL comparisons indicated the opposite distribution, with relatively more upregulated than downregulated genes (Table 1B). Altogether, these results reflect gradual metabolic reprogramming as nitrate availability decreases. Volcano plots (Figure 4 and Figure S1) illustrate these patterns and the associated fold changes (FCs) and statistical variability in gene expression.

Gene clusters with high FC and statistical significance were consistently observed. Downregulated DEGs were associated with a wide FC range in LND (Figure 4A) and LN2 comparisons against NR (Figure 4B), while upregulated genes were clustered at relatively lower FC values, with only a few DEGs scattered at a higher FC, particularly evident in NL2 vs. LND conditions (Figure 4C). These results suggest that our study may have captured early, specific transcriptional upregulation during the NL-LND transition, which may precede a more extensive upregulation, as reported for *P. cruentum* under strict nitrogen deprivation [23].

### 2.5. GO Enrichment Revealed That Nitrogen Stress Drives Metabolic Reprogramming Through Global Downregulation but Also the Upregulation of Selective Metabolic Pathways in P. purpureum

Gene Ontology (GO) results were hierarchically analyzed regarding three categories: Biological Process (BP), Molecular Function (MF), and Cellular Component (CC).

**Figure 5:**
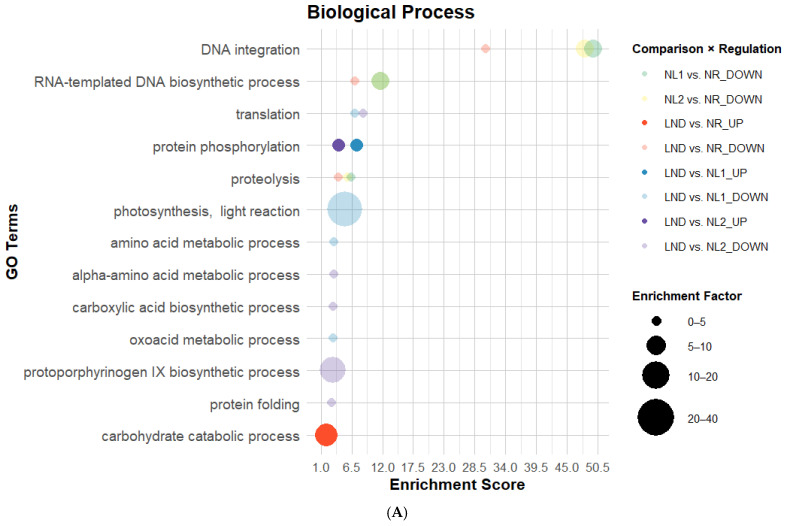

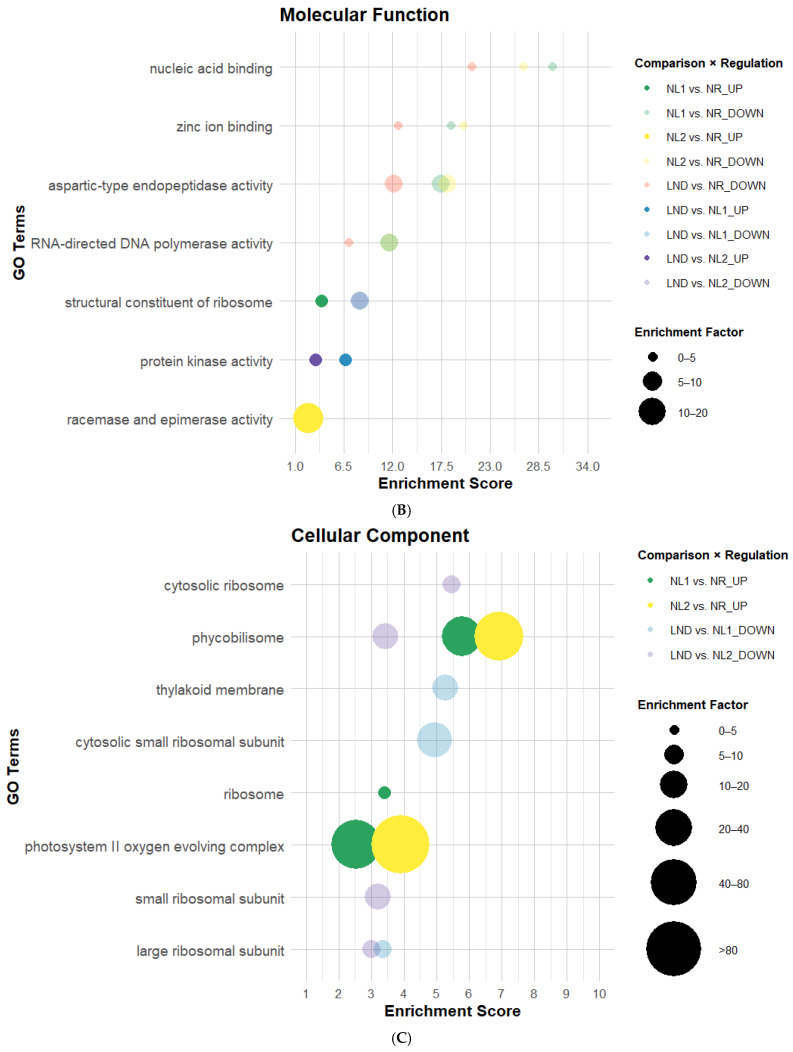
Bubble plots of enriched GO categories in DEGs across pairwise comparisons: Biological Process (**A**), Molecular Function (**B**), and Cellular Component (**C**). GO terms associated with DEGs using Blast2GO, illustrated here, possess enrichment score (ES) > 2. Bubble size indicates the Enrichment Factor (EF), illustrating the relative number of genes attributed to each GO term; color represents pairwise comparison; and opacity shows regulation direction: opaque = upregulated, transparent = downregulated (Dataset S5).

All GO categories comprised GO terms overrepresented in mostly downregulated DEGs across comparisons, which is coherent with DEG distribution (Table 1 and Figure 3 and Figure 4). Fourteen GO terms were consistently enriched in at least two pairwise comparisons, suggesting a reproducible evolution of GO enrichment across *P. purpureum* cultivation. BP-associated GO annotations (Figure 5A) had the highest GO term count and strongest enrichment scores (ES), while the Molecular Function (MF) category showed the lowest GO attribution (Figure 5B). Cellular Component (CC) terms (Figure 5C) showed the lowest ES but highest EF values. “DNA integration” showed consistent re-occurrence alongside “RNA-templated DNA biosynthetic process” (Figure 5A), “nucleic acid binding”, “zinc ion binding”, and “RNA-directed DNA polymerase activity” (Figure 5B) in NR-related comparisons. GO terms associated with DNA/RNA-related processes were predominantly enriched among downregulated DEGs. “Proteolysis” and “aspartic-type endopeptidase activity” followed a similar pattern as DNA/RNA-related GO terms within the same comparisons, which contrasted with [22]. In fact, GO terms related to LND vs. NL among downregulated DEGs also indicated significant ES and EF values related to amino acid and oxoacid metabolic processes, translation, protein folding (Figure 5A), and translation-related Cellular Components (Figure 5C). Additionally, transient upregulation of the GO term “structural constituent of ribosome” in NL1 vs. NR was followed by stronger enrichment among downregulated DEGs in LND vs. NL1. The predominance of downregulated DEGs related to protein metabolism and translation indicates that metabolic reprogramming no longer prioritizes cell growth and energy-intensive processes. This observed dynamic regulation of ribosomal structural composition, contrasting with the results from [22], may suggest the complex regulation of translational capacity under nitrogen stress.

Moreover, nitrogen stress is generally associated with photosynthesis downregulation in microalgae [22,23], which was consistent with BP-related GO term attributions “photosynthesis light reaction” and “protoporphyrinogen IX biosynthetic process” in LND vs. NL comparisons. However, EF values from 20 up to >80, seen at lower ES, were linked to “phycobilisome” and “photosystem II oxygen evolving complex” among upregulated DEGs across NL vs. NR comparisons. This result aligns with the isolated upregulation of photosystem II D1 reaction center observed by [23] and both upregulated gene coding for photosynthetic ATP production described by [22]. This may suggest that global repression of photosynthesis-related processes coexists with selective upregulation of specific photosynthetic components.

Finally, although no GO terms directly linked to nitrogen metabolism were detected at sufficient abundance or significance to generate notable EF, “protein phosphorylation”, “carbohydrate catabolic process” (Figure 5A), and “protein kinase” (Figure 5B) were uniquely overrepresented among upregulated DEGs. While enrichment of protein phosphorylation and kinase GO terms aligns with the central role of signaling processes in abiotic stress responses in plants and microorganisms [40], the upregulation of carbohydrate catabolism is less intuitive. This result may reflect the enrichment of carbohydrate catabolic functions commonly associated with central energy metabolism, while also potentially supporting carbon remobilization toward polysaccharide precursor synthesis. Altogether, GO enrichment patterns under nitrogen stress suggest an overall downregulation of growth and energy-intensive processes, alongside the selective upregulation of signaling, photosynthesis, and carbohydrate catabolism, potentially representing initial transcriptomic steps toward EPS synthesis.

KEGG enrichment analysis revealed a predominant overrepresentation of K-terms in the “Metabolism” category, particularly within “Global and overview maps”, indicating a broad shift in central metabolic processes (Figure S2 and Dataset S6). Most enriched, specific KEGG pathways (>90% of annotated K-terms) were related to amino acid, cofactor/vitamin, carbohydrate, energy, and glycan metabolism, in that respective order. Lesser enrichment was seen in lipid, nucleotide, and minor pathways. Greater carbohydrate and glycan metabolism enrichment compared to lipids align with early carbon redirection towards starch and polysaccharide synthesis rather than lipid accumulation under nitrogen stress [22,23,32]. We further focused on KEGG pathways related to nitrogen/carbon metabolism, specifically pathways known to be involved in carbon partitioning such as photosynthesis, the pentose phosphate pathway (PPP), and glycolysis/gluconeogenesis. DEGs related to at least one K-term or gene product and reflecting the highest FC were extracted from Table S2 and discussed sequentially.

### 2.6. Nitrogen Metabolism Under Early Nitrogen Limitation Suggests a Phase- and Potential-Compartment-Specific Regulation in P. purpureum

Ten DEGs were associated with K-terms linked to nitrogen uptake and assimilation, including four DEGs encoding for high-affinity nitrate transporters (NRT2.5; Table 2).

The latter showed consistent downregulation in LND vs. NR and NL vs. NR comparisons, except for one NRT2.5-coding DEG (POR6298..scf295_1), which was upregulated in LND vs. NL. Still, overall downregulation is consistent with sampling prior to conditions typically associated with high-affinity transporter NRT2.5 activation, typically induced under external nitrate concentrations < 0.06 g/L [21,41]. The remaining six DEGs encoded for key nitrogen assimilation enzymes are as follows: nitrate reductase (NaR), ferredoxin–nitrite reductase (NiR), glutamate dehydrogenase (GluDH), and glutamine synthetase (GlnS). Three NaR-associated DEGs and one NiR-associated DEG were consistently downregulated in LND vs. NR and LND vs. NL, supporting the idea that samples corresponded to a transition phase between NR and LND in our study, prior to their expected upregulation under prolonged nitrogen deprivation [32]. While GlnS was upregulated in NL2 vs. NR but strongly downregulated in LND vs. NL comparisons, GluDH showed consistent upregulation across most comparisons.

**Figure 6:**
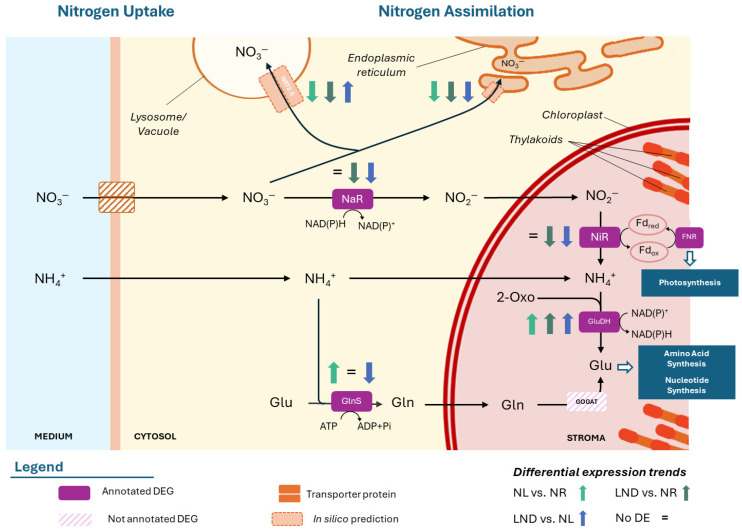
Hypothetical nitrogen uptake and assimilation pathways of *P. purpureum* under nitrate deprivation. Differential expression of KEGG Orthology (KO)-annotated genes identified in pairwise comparisons between nitrogen-replete (NR), nitrogen-limited (NL1, NL2), and late-stage nitrogen-depleted (LND) conditions. “=” represents no significant differential expression. The annotated abbreviations correspond to those used to represent the genes in Table 1. Additional information is available in Table S2.1. Blue boxes represent major metabolisms. Nitrate (NO_3_^−^), nitrite (NO_2_^−^), ammonium (NH_4_^+^), nitrate reductase (NaR), nitrite reductase (NiR), ferredoxin-NADP reductase (FNR), glutamine (Gln), glutamate (Glu), glutamine synthetase/glutamate synthase (GlnS/GOGAT), glutamate dehydrogenase (GluDH), and 2-oxoglutarate (2-Oxo). The schematic illustrations in this study were strongly inspired by [22,42].

Opposite regulation among DEGs annotated to similar gene products were noted in nitrogen metabolism but also in other targeted pathways, a pattern previously reported in the literature [22,23]. To explore this further, an in silico subcellular localization analysis was performed for DEGs sharing similar gene products with considerable FC and statistical significance (Dataset S7). Preliminary results revealed that differentially expressed isoforms were predicted to target distinct cellular compartments, suggesting the potential compartment-associated adaptation of nitrogen uptake and assimilation pathways (Figure 6).

Consistently, downregulated NRT2.5 transporters showed a putative consensus of subcellular localization association with endoplasmic reticulum (ER), whereas the punctually upregulated DEG, POR6298..scf295_1, was predicted to localize to lysosomal/vacuolar organelles. The overrepresentation of amino acid metabolism and translation among downregulated DEGs concurs with the downregulation of putative ER-associated transporters. In contrast, the transient upregulation of nitrate transporters predicted in silico to lysosome/vacuoles supports a hypothesis of vacuolar storage under pre-critical nitrogen stress, as described in diatoms [32]. Together, these results highlight the importance of the intracellular nitrogen pool and may also align with the consistent upregulation of GluDH, which could also contribute to intracellular nitrogen dynamics through early scavenging and remobilization through ammonia [32]. Nevertheless, further experimental validation of subcellular localization is crucial to confirm preliminary compartment-specific function hypotheses.

### 2.7. Dynamic Regulation of Photosynthesis and Central Carbon Metabolism Under Nitrogen Stress in P. purpureum

Understanding how nitrogen stress reshapes photosynthetic and redox metabolism is essential to explain energy redistribution and carbon partitioning toward glycolysis, the pentose phosphate pathway (PPP), and downstream processes such as EPS synthesis. In the photosynthesis pathway (Table 3), 14 DEGS were annotated to K-terms associated mainly with constitutive proteins of photosystems I (PSI), II (PSII), the photosynthetic electron transfer chain, and plastidial ATP synthase. PSII- and PSI-related DEGs showed a two-phase transcriptional pattern: upregulation from NR to NL conditions, followed by downregulation from NL to LND, the latter being coherent with expected photosynthesis regulation under advanced LND [23,32]. Although no KEGG K-term were directly associated with phycobilisomes, results aligned with the GO enrichment of PSII oxygen-evolving enhancer proteins (OEC 1,2,3). NR to NL upregulation of photosynthesis-related genes, both constitutive and functional, is consistent with a compensatory transcriptional response to maintain photosystem function. Meanwhile, downregulation from NL to LND may reflect a more severe adaptation strategy, minimizing photodamage and reactive oxygen species (ROS) generation [20,43].

PSI and PSII generate NAD(P)H and ATP via electron transport supported by cytochrome b6-f (cytb6f) and ferredoxin–NADP^+^ reductase (FNR). FNR followed the two-phase regulation of PS-related genes, whereas cytb6f-coding DEG showed consistent upregulation from NR to LND conditions [20,32]. During the NR to NL transition, cytb6f was consistently upregulated, while ferredoxin and FNR were downregulated. This pattern is coherent with a shift towards cyclic electron flow over linear electron flow, favoring ATP over NAD(P)H production, despite potential PSII damage [23].

Opposite transcriptional regulation of NAD(P)H-dependent enzymes (Figure 6: downregulated NaR-coding DEGs; upregulated GluDH-coding DEGs) suggests redox rebalancing and reducing power accumulation under early nitrogen deprivation. Although reduced linear versus cyclic electron flow has been described in *Chlamydomonas reinhardtii* [43], ATP production under nitrogen deprivation in *Porphyridium* species has been suggested to rely mainly on oxidative phosphorylation [22,23]. Consistently, nine ATP synthase/proton pump-coding genes were upregulated in our study, among other oxidative phosphorylation components (Table S2.5), suggesting the upregulation of respiratory ATP production to dissipate excess reducing power and maintain energy homeostasis. With no significant results linked to the Calvin–Benson cycle and carbon-fixation-related genes (such as RuBisCO), we propose that early nitrogen stress may primarily affect carbon partitioning through altered energy and redox balance, potentially favoring the alternative pentose phosphate pathway (PPP).

**Figure 7:**
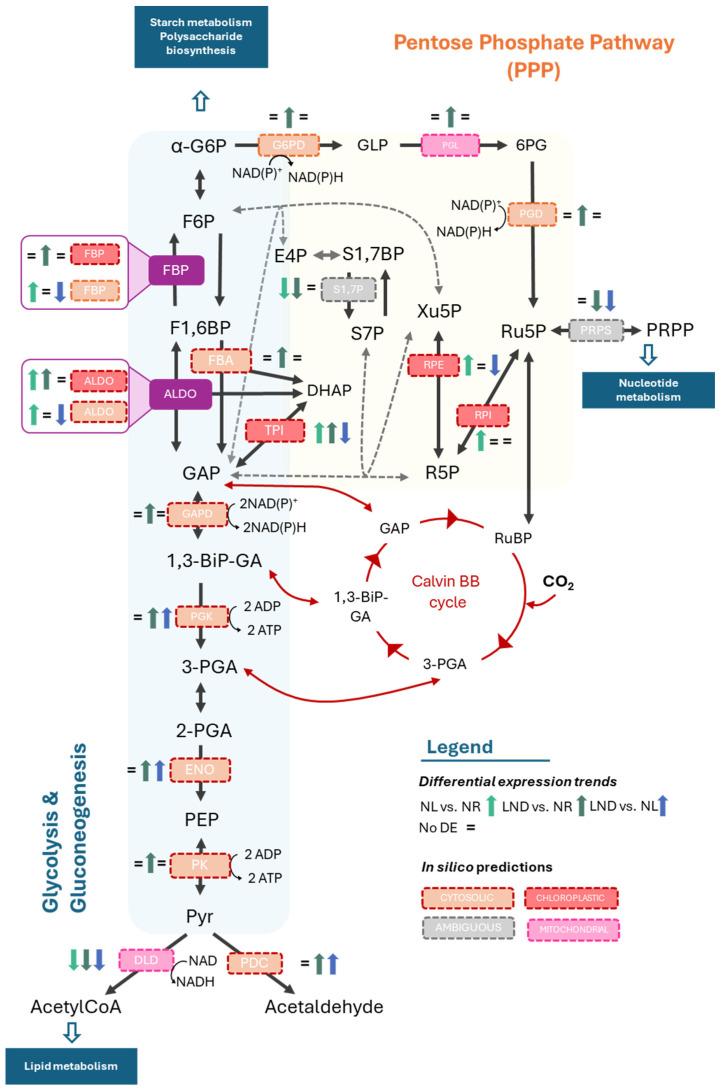
Hypothetical glycolysis, gluconeogenesis, and pentose phosphate pathway (PPP)-coordinated regulation in *P. purpureum* under nitrogen deprivation. Differential expression of KEGG Orthology (KO)-annotated genes identified in pairwise comparisons between nitrogen-replete (NR), nitrogen-limited (NL), and late-stage nitrogen-depleted (LND) conditions. “=” represents no significant differential expression. Yellow background envelopes PPP routes; blue backgrounds envelopes glycolysis and gluconeogenesis; and green background envelopes interconnecting routes. Enzymes and their putative subcellular localization predicted in silico are described in the figure’s caption. Blue boxes represent major metabolisms. Dotted gray arrows represent putative plastidial bypass of the PPP suggested by [44]. Glucose-6-phosphate (G6P); glucose-6-phosphate dehydrogenase (G6PD); phosphogluconolactonase (PGL); 6-phosphogluconate dehydrogenase (PGD); Ribulose-5-phosphate (Ru5P); Ribulose-1.5-biphosphate (RuBP); Ribose-5-phosphate (R5P); phosphoribosyl pyrophosphate synthetase (PRPS); phosphoribosyl pyrophosphate (PRPP); fructose-6-phosphate (F6P); fructose-1,6-bisphosphatase (FBP); fructose-bisphosphate aldolase (FBA); aldolase (ALDO); erythrose 4-phosphate (E4P); sedoheptulose-1,7-biphosphate (S1,7P); sedoheptulose-7-phosphate (S7P); xylulose-5-phpsphate (Xu5P); dihydroxyacetone phosphate (DHAP); glyceraldehyde-3-phosphate (GAP); glyceraldehyde-3-phosphate dehydrogenase (GAPD); 1,3-bisphosphoglycerate (1,3-BiP-GA); phosphoglycerate kinase (PGK); 3-phosphoglycerate (3-PGA); 2-phosphoglycerate (2-PGA); enolase (ENO); phosphoenolpyruvate (PEP); pyruvate kinase (PK); pyruvate (Pyr); carbon dioxide (CO_2_); dihydrolipoyl dehydrogenase (DLD); and pyruvate decarboxylase (PDC). Additional information is available in Table S2.3–S2.5. The schematic illustrations in this study were strongly inspired by [22,42].

The PPP contributes to redox balance under nitrogen stress, provides precursors for nucleotide synthesis and precursors for activated sugar donors required for polysaccharide synthesis. The PPP consists of an oxidative and non-oxidative phase, with the latter occurring alongside glycolysis/gluconeogenesis (Figure 7) and sharing intermediates such as glucose-6-phosphate (G6P), fructose-6-phosphate (F6P), and glyceraldehyde-3-phosphate (GAP), allowing for rapid metabolic adjustments and supplying routes for sugar bases used for polysaccharide synthesis [6].

In our study, G6P-accumulating enzymes (e.g., glucose phosphotransferases, G6P isomerases) were upregulated, feeding the glycolysis, gluconeogenesis, and the PP(P) pathways. Key glycolytic enzymes, such as fructose-bisphosphate aldolase (FBA), glyceraldehyde-3-phosphate dehydrogenase (GAPDH), and pyruvate kinase (PK) were generally upregulated, indicating increased glycolytic flux under nitrogen stress. In parallel, the dihydrolipoyl dehydrogenase (DLD) component of the pyruvate dehydrogenase complex was downregulated, alongside significant upregulation of the pyruvate decarboxylase (PDD), suggesting a metabolic redirection from acetyl-CoA toward alternative intermediates.

Interestingly, mixed regulation among DEG isoforms bridging glycolytic intermediates and the PPP were observed. Overall, enzyme-encoding DEGs of the upper glycolytic pathway were predicted in silico to the chloroplast, while those in the lower pathway were predicted to the cytosol. This result aligns with observations in *C. reinhardtii* [45], encouraging further investigation on enzymatic compartmentalization in this red microalga. In silico predictions exhibited compartment-dependent expression patterns of fructose-1,6-bisphosphatase (FBP) and aldolase (ALDO)-coding genes. Putative plastidial isoforms were primarily upregulated, steering metabolism towards gluconeogenesis and PPP intermediates (F6P and dihydroxyacetone phosphate (DHAP), respectively). In contrast, the corresponding cytosolic isoforms were downregulated towards LND conditions, favoring glycolytic processes. DEG-encoding cytosolic FBP was upregulated from NR to NL conditions but subsequently downregulated from NL to LND. This pattern may indicate a transient activation of gluconeogenic flux in the cytosol during early nitrogen stress, followed by a shift toward glycolytic flux in LND, which is consistent with previous studies [22,32]. In parallel, PPP-related genes such as PGD, phosphoribosyl pyrophosphate synthetase (PRPS), and putative plastidial ribulose-phosphate epimerase (RPE) were downregulated in LND stages, while putative cytosolic glucose-6-phosphate dehydrogenase (G6PD) and 6-phosphogluconate dehydrogenase (PGD) were predominantly upregulated from NR to LND.

To summarize, our results indicate that putative cytosolic glycolytic and PPP-related genes were predominantly upregulated from NR to LND conditions, whereas putative plastidial PPP and glycolytic enzymes showed downregulation under advanced nitrogen depletion. This pattern supports a transient activation of gluconeogenesis during the early stages of nitrogen deprivation [22], followed by a redistribution of carbon flux toward NADPH- and ATP-generating pathways to sustain energy and redox balance under prolonged stress. Several PPP intermediates can feed into the nucleotide metabolism and the formation of activated sugar donors, linking central carbon metabolism to downstream polysaccharide production. However, PPP-intermediates that can be converted into glycolytic intermediates (e.g., R5P, Xu5P) through transketolase reactions, and therefore participate in carbon partitioning, were not elucidated through our DEG and KEGG enrichment analysis. This once again highlights the need to experimentally resolve enzyme compartmentalization expression.

### 2.8. Concerted Regulation of Nucleotide–Sugar Synthesis and CAZyme-Related Pathways Supports Early Polysaccharide and Glycoprotein Assembly Under Nitrogen Stress

**Figure 8:**
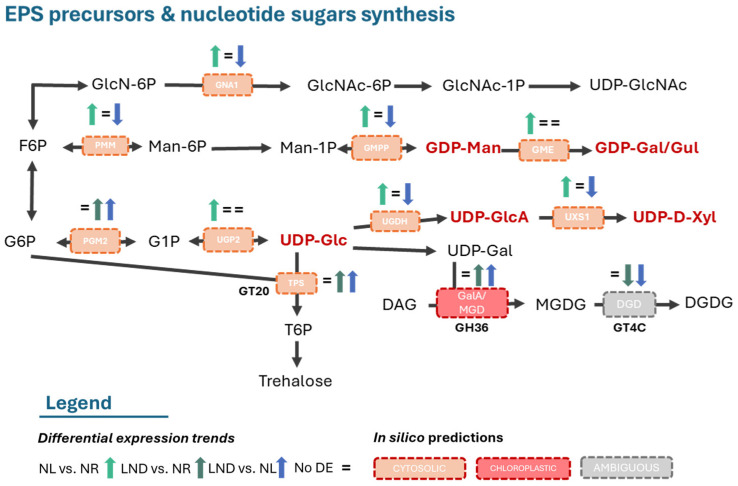
Hypothetical EPS precursors and nucleotide–sugar synthesis. Differential expression (DE) of K-term-annotated genes identified in pairwise comparisons. GT20, GH36, and GT4C description in Figure 9. Additional information is available in Table S3.1–S3.2. The schematic illustrations in this study were strongly inspired by [22,42].

To further link the early activation of gluconeogenesis to the onset of polysaccharide synthesis, KEGG enrichment analyses were conducted for nucleotide–sugar and amino–sugar biosynthesis pathways (Figure 8 and Table S3). Overall, nucleotide–sugar synthesis was mainly upregulated from NR to NL conditions and downregulated from NL to LND. Among the glycosyl donors reported by [22], we identified five enzyme-coding genes directly responsible for precursor synthesis, primarily belonging to the UDP- and GDP- sugar families (UDP-Glc, UDP-GlcA, UDP-D-Xyl, GDP-Man, and GDP-Gal/Gul). DEGs were annotated as phosphoglucomutase/phosphopentomutase (PGM2) and UTP-α-D-glucose-1-phosphate uridylyltransferase (UGP2), both showing overall upregulation of the UDP-Gluc synthesis pathway. Although both enzymes catalyze reversible reactions, early upregulation of UDP-glucose 6-dehydrogenase (UGDH)- and UDP-xylose synthase (UXS1)-coding genes, responsible for UDP-GlcA and UDP-Xyl production, support UDP-Gluc production as their precursors. Similarly, early upregulation of GDP-mannose pyrophosphorylase (GMPP), leading to GDP-Man synthesis, is coherent with the hypothesis that phosphomannomutase (PMM) and even GDP-mannose 3,5-epimerase (GME) show upregulation towards the biosynthesis of such GDP-sugars, including GDP-Gal/Glc. Additionally, early upregulation of GNA1, which involves the irreversible enzyme-catalyzing production of GlcNAc-6-P, suggests potential upregulation of UDP-GlcNAc biosynthesis.

Altogether, the KEGG enrichment and DEG analyses under nitrogen stress at the transcriptomic level are consistent with the general monosaccharide composition of *P. purpureum* EPS, including xylose, glucose, GlcA, and GalA, supporting the notion of a conserved polysaccharide backbone and a coordinated activation of nucleotide–sugar biosynthesis pathways during the early stages of nitrogen limitation.

Following the synthesis of activated sugars, these sugar donors serve as substrates for the biosynthesis of disaccharides, highlighting the interconversion of hexoses to disaccharides. Non-reducing disaccharide, trehalose, was suggested to be the initial step of polysaccharide synthesis by [22]. One trehalose-6-phosphate synthase (TPS)-coding DEG, responsible for the generation of trehalose-6-phosphate (T6P) using UDP-Gluc and G6P, was indeed identified in our study and was upregulated during early nitrogen deprivation.

No trehalose-6-phosphate phosphorylase (TPP)-coding DEGs, leading to the direct synthesis of trehalose, were identified. Although trehalose and its precursor T6P are difficult to analyze due to low abundance in plants, both have been implicated in diverse regulatory roles coordinating multiple metabolic processes [46]. Regarding carbohydrate metabolism, T6P has been reported as a sugar-signaling molecule responsible for the activation of ADP-glucose pyrophosphorylase (AGPase), a controlling enzyme of the starch metabolism. Indeed, enzyme-coding DEGs related to glycogen/starch metabolism were identified, such as glycogen phosphorylase (PYG) and granule-bound starch synthase (WAXY). While PYG displayed overall upregulation, WAXY showed early upregulation followed by downregulation, inferring indirectly an additional activated sugar: ADP-Glc. These results may indicate an early activation of carbon storage towards amylose synthesis and subsequent Floridian starch accumulation, the primary energy storage in *Porphyridium* species [5,30,35]. In red algae, α-D-galactopyranosyl-(1-2)-glycerol (floridoside) has been proposed as a precursor for cell wall polysaccharides in *Porphyridium* sp. [47]. In *Galdieria sulphuraria* [48], TPS-like enzymes were annotated but functioned as (iso)floridoside phosphate synthases. No UDP-glucose 4 epimerase (GALE)-coding DEG directly involved in UDP-Gal and subsequent floridoside synthesis was identified in our study. However, late upregulation of one DEG annotated to multiple K-terms associated with GalA and MGD activities suggests α-linked galactose-hydrolysis, indicating the possible presence of this activated sugar. These observations highlight the need for the functional validation of TPS-annotated genes in *Porphyridium* sp. Altogether, these results indicate that nitrogen stress may promote the early upregulation of nucleotide and activated sugar biosynthesis, generating substrates required for polysaccharide and glycoprotein biosynthesis and assembly, known to be mediated by carbohydrate-active enzymes (CAZymes).

CAZymes are protein families involved in the synthesis, modification, and degradation of complex carbohydrates and glycans, classified into six main categories: glycoside hydrolases (GHs), glycosyltransferases (GTs), polysaccharide lyases (PLs), auxiliary activity (AAs) enzymes, carbohydrate esterases (CEs), and carbohydrate-binding modules (CBMs). In *Porphyridium purpureum*, 116 CAZymes (31 GHs, 83 GTs, and 2 CEs) and 40 putative CBMs were identified in the first genome assembly [24]. More recent transcriptomic analyses [22] reported the absence of annotated PLs and variable numbers of AAs, CEs, and CBMs homologs, reflecting incomplete or inaccurate gene predictions and further highlighting the need to refine genomic and transcriptomic annotations in this species. In our study, 91 CAZyme-coding genes were identified, predominantly GTs and GHs (Dataset S8), with 21 GT subfamilies and 12 GH subfamilies represented. The predominance of GTs (>60% of all annotated CAZymes) was not surprising given the known high diversity of GT families, potentially contributing to the biochemical complexity of *P. purpureum*’s cell wall polysaccharides compared with other red algae such as *Cyanidioschyzon merolae* [24].

**Figure 9:**
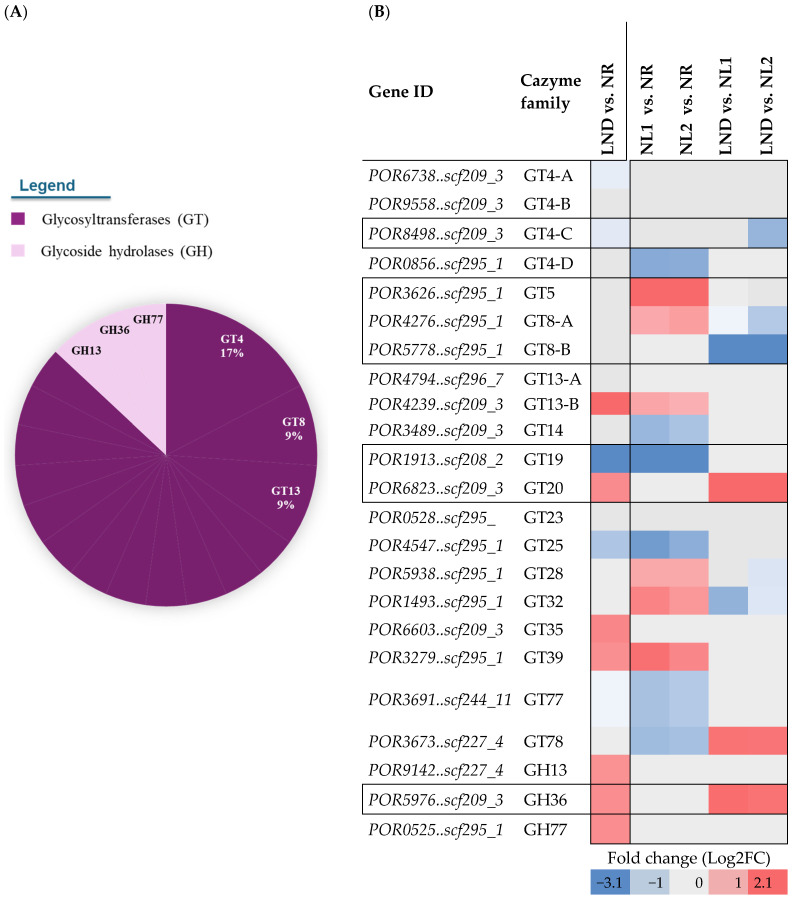
CAZYmes subfamily (**A**) distribution among identified DEGs (**B**) and their corresponding differential gene expression across pairwise comparisons. (**A**) CAZyme subfamilies representing ~35% of CAZyme distribution are tagged. All other CAzymes are associated with 1 DEG in all other subfamilies, including GT5, GT14, GT19, GT20, GT23, GT25, GT28, GT32, GT35, GT39, GT77, and GT78 (dark purple); GH13, GH36, and GH77 (light purple). (**B**) Differential expression color gradient was adjusted to max and min values of the overall table (blue: downregulated; red: upregulated). Letters A to D were attributed to GT4 genes to represent the following GenBank annotations: GT4-A, “putative glycosyltransferase”; GT4-B, “Capsular polysaccharide biosynthesis glycosyltransferase CapM”; GT4-C, Digalactosyldiacylglycerol synthase 2, chloroplastic; GT4-D, putative glycosyltransferase; and GT8-A and B, “glucoside xylosyltransferase 2”.

Under nitrogen stress, 23 CAZyme-coding genes were differentially expressed, with >80% belonging to GT families (Figure 9A and Dataset S9), alongside three GH subfamilies (GH13, GH36, and GH77). GT4, GT8, and GT13 accounted for more than one-third of all differentially expressed CAZymes, which is consistent with the mobilization of glycosidic bond-forming enzymes during nitrogen stress [22]. GT4 DEG predominance aligns with its basic constitutive role in forming α-glucosidic bonds from diverse sugar substrates [49], while repeated GT8 and GT13 annotations could reflect broader sugar substrate specificities or poorly characterized functions within polysaccharide biosynthesis [50].

Among the 23 CAZyme-related DEGs, 11 were consistently downregulated. Downregulation was observed for GT4A/B/C, GT8B, and GT23 under LND vs. NL conditions and for GT4D and GT19 during the early LND stage. While GT4A and GT4D were annotated as putative GTs, GT19, a lipid A disaccharide synthase-like homolog (lpxB), has been associated with mannosylation reactions in lipid-like oligosaccharide synthesis [50]. Early downregulation of GT19 may indicate that mannose-based sugar donors are redirected toward polysaccharide and protein-based glycan synthesis in the initial stages of nitrogen stress. This aligns with GDP-Man-dependent GT78, annotated as a mannosylglycerate synthase, being downregulated from NL vs. NR and upregulated from LND vs. NL, as well. Additionally, GT13B and GT35, encoding α-amylase and glycogen phosphorylase, respectively, suggest the enhanced mobilization or accumulation of polysaccharide precursors such as glucose-1-phosphate (G1P). GH13 and GH77, known to catalyze the cleavage and rearrangement of α-1,4 glycosidic bonds in starch-like glycans, were also upregulated.

Five CAZyme-coding DEGs displayed dynamic regulations, being upregulated under NL vs. NR and downregulated under LND vs. NL. These included GT5, GT8A, GT28, and GT32, which are involved in the synthesis of mannosyl-, xylosyl-, and glycosyl-sugar donors. This could be evidence of transient activation of polysaccharide synthesis in the early stages of nitrogen stress. Moreover, the contrasting early downregulation of GT77, annotated as an arabinosylfuranosyltransferase, with the late downregulation of GT4B, annotated as a GT involved in Capsular-like (CapM) polysaccharide biosynthesis, can point out cell wall biosynthesis and subsequent EPS release’s fine regulation. Altogether, the predominance of GT families, together with the dynamic regulation of sugar-donor-associated CAZymes (e.g., GT5, GT32, and GT78), aligns with the conserved monosaccharide composition of *P. purpureum* EPS. Specifically, the transcriptional patterns support the incorporation of xylose, glucose, galactose, and uronic acids into the polysaccharide backbone, consistent with the EPS profile reported in Section 3.2.

Seven of the 23 CAZyme-coding DEGs were functionally associated with six KEGG terms (Figure 9B): GT4C (DGD), GT8A/B (GXYLT), GT19 (lpxB), GT20 (TPS), GH13 (WAXY), and GH36 (GalA/MGD). Altogether, this points out differentially expressed CAZymes involved in multiple pathways such as glycerolipid metabolism [30], in addition to polysaccharide and glycan metabolism.

**Figure 10:**
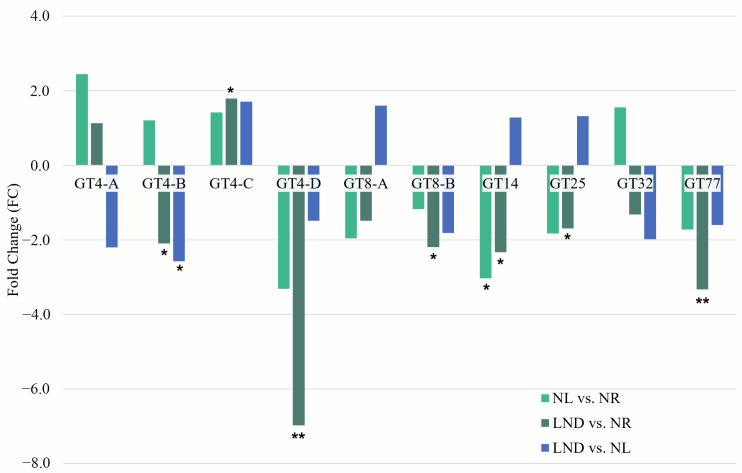
Fold change (FC) of differentially expressed glycosyltransferase measured by RT-qPCR. Differential expressions identified in pairwise comparisons: NL vs. NR (light green), LND vs. NR (dark green), and LND vs. NL (blue). Expression was normalized to the ARNr16S housekeeping gene. Relative quantification and fold changes were calculated using the 2^(−ΔΔCt) method [51]. Asterisks indicate statistically significant differences from control conditions (*, *p* < 0.05; ** *p* < 0.01 calculated using Kruskal & Wallis and Wilcoxon statistical tests).

qRT-PCR validation was performed on more than 30 GT-coding genes selected from [24] (Table S4). Among these, 10 corresponded with differentially expressed CAZymes identified by RNA-Seq (Figure 10). Overall, qRT-PCR expression patterns were consistent with RNA-Seq, such as GT4-D, GT8B, and GT77, showing a reproducible and significant downregulation. Dynamic regulation of GT32, annotated as an initiation-specific α-1.6-mannosyltransferase, was also confirmed between qRT-PCR/RNA-Seq results.

While GT4A/B only partially aligned with RNASeq trends, the upregulation of GT14 and GT25, a putative glycosaminoglycans transferases associated with (in)active glycan elongation [50], may be evidence of the later steps of polysaccharide or glycan modification and branching.

KEGG enrichment also highlighted CAZymes involved in polysaccharide polymerization through glycosylation and branching modification (e.g., GH CAZymes) but also glycan assembly pathways. The overall upregulation of ALG5, involved in the transfer of glucose from UDP-glucose to dolichol phosphate (Dol-P), in the early steps of glycan assembly aligned with the early and overall upregulation of OSTC and RPN2, essential components of the OST complex responsible for the transfer of oligosaccharides from dolichol to Asn residues and further glycan transfer from lipid-linked donors to proteins. This could be evidence of the glycoprotein synthesis required for the later steps involving cell wall biosynthesis and the consequent extracellular release of polysaccharides. KEGG enrichment also highlighted CAZymes involved not only in polysaccharide polymerization through glycosylation and branching modification (e.g., GH CAZymes) but also glycan assembly pathways (Table 4).

To summarize, differentially expressed CAZymes under nitrogen stress suggest a transcriptional program that maintains the conserved monosaccharide composition of EPS while modulating polysaccharide synthesis and remodeling according to nitrogen availability.

## 3. Materials and Methods

### 3.1. Culture Conditions and Procedure

*Porphyridium purpureum* strain 1380/1A was purchased from the Culture Collection of Algae and Protozoa (CCAP, https://www.ccap.ac.uk/ accessed on 11 January 2026) and maintained in modified Provasoli medium [34] with an N/P ≈ 4 to optimize biomass (3 g L^−1^) and exopolysaccharide (EPS) production, with the following composition per liter: 28 g NaCl, 0.73 g NaNO_3_, 0.32 g K_2_HPO_4_, 7.20 g MgSO_4_·7H_2_O, 1.55 g CaCl_2_·2H_2_O, 100 µL of 10× trace metal solution (MnSO_4_·H_2_O, (NH_4_)_6_Mo_7_O_24_·4H_2_O, CoSO_4_·7H_2_O, CuSO_4_·5H_2_O, ZnSO_4_·7H_2_O, and Fe-EDTA), and 100 µL of 10× vitamin solution (vitamin B12, thiamin, and biotin). Three independent 4 L cultures were inoculated with 500 mL of preculture. Five-liter cylindrical photobioreactor set at pH 8, 22.5 °C, cultivated cultures under continuous illumination (158 µmol/m^2^/s) and agitation (150 rpm) with air and CO_2_ set to 100 mL/min, 1004 ppm CO_2_. Samples were taken every 2–3 days for growth monitoring, and recovered supernatants (10,000× *g*, 10 min) were collected for biochemical assays. Additional large-volume samples were taken at different kinetic points to recover the algal biomass pellet for RNA extraction.

### 3.2. Growth Monitoring

#### 3.2.1. Cell Counting

Samples were taken every 2–3 days to assess cell concentration (number of cells per mL of culture) using a Malassez counting chamber. To facilitate cell counting and break aggregates, samples were pre-diluted in 28 g/L NaCl solution and sonicated (3 × 15 s, 0.4 cycles, and amplitude 60%) when necessary. No technical replicates were included for cell counting to respect volume limitation required for RNA-Seq sampling.

#### 3.2.2. Nitrate Consumption Determination

Residual nitrates in the culture medium were measured using the Cawse method, modified according to the method of [52], modified by [53]. A standard curve from 0 to 0.1 g/L was prepared from a 0.5 g/L NaNO_3_ stock solution. The reagent used was 5% perchloric acid. Absorbance was recorded at 210 nm and 275 nm. Nitrites absorb at 275 nm, allowing for correction of the final absorbance value. Sample OD was calculated as described below, and nitrate concentration (g/L) was deduced from the standard curve:

OD_sample_ = OD_210nm_ – 2 × (OD_275nm_ − ΣOD_275nm_ standard curve) (1)

#### 3.2.3. Total Sugar Determination

Preliminary desalting was performed to avoid interference from medium components during polysaccharide recovery. Samples were desalted by successive centrifugations (5000× *g*, 15 min) with water decreasing in salinity, followed by filtration through sintered glass funnels (porosity grades 1–3; 100–15 µm). The filtrate was further purified by tangential ultrafiltration (Vivaflow 200, 10 kDa cutoff; Sartorius, Göttingen, Germany), as described by [9]. Retained polysaccharides were lyophilized, resuspended in 5 mL of water, and quantified using the phenol–sulfuric acid method [54], modified as previously described [55], in which a yellow–orange color develops proportionally to total sugar content. A glucose standard curve (0.02–0.1 g/L) was prepared from a 0.1 g/L stock solution. Absorbance was measured at 483 nm, corrected with the dilution factor (DF) from the desalting step, and total sugar concentration was calculated as

Total sugar (g/L) = OD_483nm_ × DF_Desalting step_ (2)

### 3.3. Exopolysaccharide (EPS) Characterization

#### 3.3.1. Sulfate Content Determination

Sulfate content, reflecting polysaccharide sulfation, was determined by turbidimetric BaCl_2_/gelatin assay [56]. Standard curves (0.25–3 mg/mL K_2_SO_4_) were prepared, and absorbance was measured at 550 nm. Results were expressed in SO_4_ equivalent.

#### 3.3.2. Neutral and Acidic Sugar Content Determination

Neutral sugars were measured with resorcinol [55] and acidic sugars with meta-hydroxybiphenyl (m-HBP) [57]. Hot acid hydrolysis releases sugar monomers, which form furfural derivatives that condense with phenolic reagents. Absorbances were measured at 450 nm (neutral) and 520 nm (acidic). Standard curves of glucose and glucuronic acid (0.05–0.45 g/L) were used to correct cross-interference using the formula from [58].

#### 3.3.3. High-Performance Anion-Exchange Chromatography with Pulsed Amperometric Detection (HPAEC-PAD)

HPAEC was used to identify and quantify monosaccharides after acid hydrolysis of freeze-dried polysaccharides, using an ICS 3000 (Dionex, Sunnyvale, CA, USA) equipped with pulsed amperometric detection (PAD) and AS 50 autosampler, as previously described [38,39]. Hydrolysates (10 mg in 1 mL trifluoroacetic acid, 1.5 h, and 120 °C) were neutralized, centrifuged, diluted (1/10, 1/100, and 1/1000), filtered (0.22 μm), and injected on a CarboPac PA1 column (4 × 250 mm) with precolumn (4 × 50 mm). Injection volume was fixed at 25 µL. Before each injection, columns were equilibrated by running for 15 min with 18 mM NaOH. Samples were eluted isocratically with 18 mM NaOH for 25 min, followed by a linear gradient between 0 and 0.5 M sodium acetate in 200 mM NaOH for 20 min to elute acidic monosaccharides. Run was followed by 15 min washing with 200 mM NaOH. The eluent flow rate was kept constant at 1 mL/min. Columns were thermostated at 25 °C. External standards (l-Rha, l-Fuc, l-Ara, d-Xyl, d-Man, d-Gal, d-Glc, d-GlcA, and d-GalA) and internal standard addition allowed for quantitative determination of monosaccharide composition. Data were collected and analyzed with Dionex Chromeleon 6.80 software (Dionex, Sunnyvale, CA, USA).

### 3.4. Gene Expression Analysis

#### 3.4.1. RNA Extraction

Five technical replicates were collected at four kinetic timepoints associated with three nitrogen availability conditions determined through cell growth performance, residual nitrate concentrations, and total sugar concentrations. Nitrogen-replete (NR) conditions aligned with exponential growth (low sugar, high nitrate), nitrogen-limitation (NL) conditions aligned with the onset of total sugar accumulation (rising sugar content), and late-stage nitrogen depletion (LND) conditions aligned with advanced stationary phase (high sugar, low nitrate). Before RNA extraction, samples were grounded manually in liquid nitrogen. Total RNAs were extracted using the TRIzol^®^ protocol (Qiagen) according to the provider’s recommendations. After homogenizing approximately 100 mg of algal cells, 1 mL of TRIzol^®^ was added and incubated at RT for 5 min. Then, 0.2 mL chloroform per 1 mL of TRIzol^®^ used was added. After centrifugating (12,000× *g*, 15 min, and 4 °C), the upper aqueous phase containing RNAs was collected. Then, total RNA was precipitated using 0.5 mL isopropanol per 1 mL of TRIzol^®^ used in the aqueous phase. After centrifugation, the pellet was washed with 1 mL of 75% ethanol per 1 mL of original TRIzol^®^. The pellet was dried and resuspended in 20 µL of RNAse-free water for further analyses. Extracted RNA integrity was verified on 1% agarose gels.

#### 3.4.2. Gene Selection, Primer Design, Reverse Transcription, and Quantitative PCR (RT-qPCR)

Primers targeting 33 genes belonging to the glycosyltransferase (GT) family were selected to perform qRT-PCR (Table S5). These genes were chosen from data published by [24]. Primers were designed using Primer3plus software v.3.2.0, setting a fragment size between 280 and 320 bp. A temperature gradient assay was performed by PCR to determine the hybridization temperature for quantitative PCR (qRT-PCR) (60 °C, 63.8 °C, and 68 °C), and ARNr16S gene, as a housekeeping gene, was designed based on sequence alignment and synthesized by Eurofin genomics [https://eurofinsgenomics.eu, accessed on 1 January 2022]. Reverse transcription (RT) of 2 µg of total RNAs was carried out using the Maxima H minus first strand cDNA synthesis kit with dsDNAse (Thermofisher K1682), following the manufacturer’s protocol for the provided instructions (DNAse: 2 min 37 °C, Rt: 30 min 50 °C, and 5 min 85 °C). RTqPCRs were conducted on 1:20 diluted cDNAs on the Gentyane genotyping platform [https://gentyane.clermont.inrae.fr/, accessed on 1 January 2022], using a Hamilton Genomics Starlet robot to distribute the cDNAs and the Roche Green 1 Master mix (04887352001). RTqPCR was performed in a Roche 480 Light Cycler (95 °C 10 min, 45 amplification cycles (95 °C 10 s, 60 °C 15 s, and 72 °C 15 s) and melting curve (95 °C 15 s, 60 °C 15 s, and 95 °C continuously with acquisition every 5 °C)). Results were analyzed using the LightCycler480SW1.5 software, normalized by the housekeeping gene, and then processed using the 2−ΔΔCt method described by [51].

### 3.5. Transcriptomic Analysis

#### 3.5.1. RNA-Seq Procedure and Analysis

RNA Sequencing was carried out on the Genotoul platform in Toulouse (France) using one channel. Stranded mRNA libraries were prepared using the Illumina TruSeq Stranded mRNA Library Preparation Kit, following the manufacturer’s instructions. Libraries were pooled and sequenced using an SP flow cell. Paired-end sequencing was performed, generating reads of 150 base pairs from each end (2 × 150 bp) with standard NovaSeq chemistry.

#### 3.5.2. Mapping Reads to *Porphyridium* Genome

Mapping reads to reference sequences was performed using rna_star 2.7.8a with default parameters on the Galaxy platform. The genome assembly and annotation version of *P. purpureum* used as reference in this study has been published by [27] and downloaded from http://porphyra.rutgers.edu (accessed on 1 April 2022). Raw read counts were created using featurecounts 2.0.1 on the Galaxy web server. The annotation version 2 of *P. purpureum* genome (file Porphyridium_purpureum_v2_genemodels.gff, downloaded from http://porphyra.rutgers.edu, accessed on 1 April 2022) was used to estimate the raw expression of each gene (feature type filter = gene).

#### 3.5.3. Principal Component and Differential Expression Analyses

Principal component analysis (PCA) was carried out on variance-stabilized transformed (VST) counts. This transformation was applied to stabilize variance across the dynamic range of expression values and to allow for an unbiased exploration of sample clustering. Differential expression analysis was performed using DESeq2 (v1.34.0) as implemented on the DEBrowser platform (DEBrowser v1.22.5, [59]). Raw count data were normalized using the median-of-ratios method, and dispersion parameters were estimated according to the negative binomial distribution model. To identify differentially expressed genes (DEGs), the likelihood ratio test (LRT) framework was applied. Resulting *p*-values were corrected for multiple testing using the Benjamini–Hochberg method to control the False Discovery Rate (FDR, adjusted *p*-value). FDR was assigned an adjusted *p*-value cutoff of 0.01, and no log_2_ fold-change (FC) shrinkage was applied in this analysis. Missing values (NA) were taken out, and all genes and samples had verified variances (variance > 1^−10^) to ensure consistency and homoscedasticity across all samples.

#### 3.5.4. Gene Ontology (GO) Enrichment Analysis

GO functional enrichment analysis was performed using Blast2GO. Following best practice recommendations for input gene subsetting [60], GO terms assignment was performed separately for upregulated and downregulated differentially expressed genes (DEGs) in each pairwise comparison. For each GO term identified as overrepresented, the proportion of GO-annotated DEGs associated with a given GO term (GeneRatio) was calculated as

GeneRatio = n° DEGs annotated to the GO term/Total n° GO-annotated DEGs(3)

GeneRatio was contextualized relative to the frequency of the same GO term in the GO-annotated gene set to assess overrepresentation compared to expectation by chance. This was considered by calculating the Enrichment Factor (EF) as

Enrichement Factor (EF) = GeneRatio/(n°genes annotated to the GO term/Total n° GO-annotated genes)(4)

Finally, the significance of FDR values, expressed as enrichment score, was calculated as

Enrichment score (ES) = −log_10_(FDR) (5)

GO terms with statistically significant enrichment (−log_10_(FDR) > 2.0; FDR < 0.01) were represented in bubble plots using RStudio v.2025.09.0+387. FDR significance, expressed as −log_10_(FDR), was assessed to evaluate the robustness of both common and uniquely enriched terms in pairwise comparisons.

#### 3.5.5. KEGG (K-Term) Enrichment Analysis

KEGG (Kyoto Encyclopedia of Genes and Genomes) database used to perform K-term annotations of differentially expressed genes (DEGs) across pairwise comparisons using BlastKOALA. CAZyme-coding DEG with BlastKOALA K-term annotations were completed with KofamKOALA and GhostKOALA analyses, each being run in duplicates. Final assignments were kept when multiple tools pointed towards a “consensus” annotation, and single annotations were also retained if their scores were above the defined threshold for the respective K-term.

### 3.6. In Silico Subcellular Localization Prediction Analysis

DEGs linked to common K-terms and showing opposite FC patterns within the same sampling timepoint were complemented by in silico subcellular localization prediction using three servers: TargetP 2.0, DeepLoc 2.0/2.1, and LOCALIZER 1.0.4. “Consensus” subcellular localization was assigned when at least two tools provided the same output, whereas mutually exclusive predictions were classified as “ambiguous.” Probability thresholds of DeepLoc 2.0/2.1 prediction tool are listed in Supplementary Materials. TargetP 2.0 was run using the “Plant” organism filter [61]. Multi-label predictor DeepLoc 2.0/2.1 was used with the “High throughput (Fast)” model and “long output” format [62]. LOCALIZER predictions were performed with sequences designated as “Full plant sequences” [63].

## 4. Conclusions and Perspectives

Under progressive nitrogen stress, *P. purpureum* revealed a gradual, phase-dependent metabolic response characterized by temporally structured metabolic shifts rather than a binary transition to deprivation. Physiological characterization showed that reduced nitrogen uptake coincided with polysaccharide secretion, linking carbon overflow and EPS release to external nitrogen availability. Transcriptomic analyses indicated global metabolic downregulation and a selective upregulation of stress-responsive functions related to photosystem composition, redox homeostasis, and carbohydrate catabolism, hinting at metabolic redirection towards nucleotide–sugar metabolism. Upregulation of the carbohydrate catabolic metabolism and nucleotide–sugar synthesis, aligned with the coordinated induction of specific CAZyme families (GT4, GT8, and GT77), supports a tightly regulated balance between polysaccharide synthesis/polymerization and glycan assembly.

Our findings highlight enzyme compartmentalization as a key research priority in polysaccharide metabolism, as preliminary in silico predictions indicate phase- and compartment-specific nitrogen metabolism during early nitrogen stress [32,44]. Experimental validation and future cluster-based transcriptomic analyses may further resolve coordinated regulatory responses in *Porphyridium* spp. [64].

## Figures and Tables

**Table 1:** Differentially expressed genes (DEGs) across nitrogen availability transitions in *P. purpureum* pairwise comparisons. (A) Pairwise comparisons relative to nitrogen-replete (NR) conditions, NL1 vs. NR, NL2 vs. NR, and LND vs. NR; and (B) pairwise comparisons within nitrogen-limited conditions, LND vs. NL1 and LND vs. NL2, are shown. Up- and downregulated DEGs are shown, with total DEGs reported for each comparison. Further details can be found in Table S1.

A				
Comparison	Condition	Upregulated	Downregulated	Total DEGs
NL vs. NR	NL1	550	732	1282
	NL2	608	786	1394
LND vs. NR	-	683	1004	1687
**B**				
**Comparison**	**Condition**	**Upregulated**	**Downregulated**	**Total DEGs**
LND vs. NL	NL1	541	453	994
	NL2	586	539	1125

NR, nitrogen-replete; NL1, nitrogen-limited (timepoint T2); NL2, nitrogen-limited (timepoint T3); and LND, late-stage nitrogen-depleted (timepoint T4).

**Table 2:** Differential gene expression of K-term-associated DEGs related to nitrogen metabolism in *P. purpureum* (extract from Table S2.1). Differential expression of KEGG Orthology (KO)-annotated genes identified in pairwise comparisons between NR, NL (1-2), and LND conditions, expressed in log_2_ fold change (FC). Differential expression color gradient was adjusted to max and min values of the overall table. Gray cells indicate genes that were not differentially expressed or not detected in the corresponding comparison The annotated abbreviations correspond to those used to represent the genes in Figure 6.

		Fold Change (FC)				
Gene ID	Annotation (Abbreviation)	LND vs. NR	NL1 vs. NR	NL2 vs. NR	LND vs. NL1	LND vs. NL2
*POR6298..scf295_1*	High-affinity nitrate transporter 2.5 (NRT2.5)		−1.9	−1.7	2.9	2.7
*POR8948..scf295_1*	High-affinity nitrate transporter 2.5 (NRT2.5)	−4.4	−1.8	−1.7	−2.5	−2.6
*POR1427..scf295_1*	High-affinity nitrate transporter 2.5 (NRT2.5)	−3.8	−2.2	−2.4	−1.5	−1.3
*POR7993..scf295_1*	High-affinity nitrate transporter 2.5 (NRT2.5)	−1.4				
*POR6787..scf295_1*	Nitrate reductase NADH (NaR)	−2.7			−2.9	−2.8
*POR8251..scf295_1*	Nitrate reductase NADH (NaR)	−2.9			−2.7	−2.7
*POR3077..scf295_1*	Nitrate reductase NADH (NaR)	−1.2				
*POR3485..scf295_1*	Glutamate dehydrogenase (GluDH)	2.0		1.2	1.0	
*POR2445..scf236_6*	Ferredoxin–nitrite reductase (NiR)	−1.5			−2.3	−2.4
*POR7605..scf227_4*	Glutamine synthetase isozyme (GlnS)			1.2	−1.1	−1.6

**Table 3:** Differential gene expression of K-term-associated DEGs related to photosynthesis in *P. purpureum* photosynthesis (extract from Table S2.2). Differential expression of KEGG Orthology (KO)-annotated genes identified in pairwise comparisons between NR, NL (1-2), and LND conditions, expressed in log_2_ fold change (FC). Differential expression color gradient was adjusted to max and min values of the overall table.

		Fold Change (FC)				
Gene ID	Annotation (Abbreviation)	LND vs. NR	NL1 vs. NR	NL2 vs. NR	LND vs. NL1	LND vs. NL2
*POR6298..scf295_1*	PS II Psb27		2.0	2.2		−1.2
*POR3050..scf295_1*	PS II oxygen-evolving enhancer (OEC)		2.3	2.7	−1.5	−1.9
*POR2665..scf244_11*	Cytochrome b 6-f complex subunit	1.1	1.1	1.2		
*POR2912..scf227_4*	Photosystem I subunit psaO		2.5	2.4	−1.7	−1.5
*POR6098..scf229_5*	Chlorophyll a-b binding protein/PSI-LHC		2.5	2.9	−1.5	−1.9
*POR8201..scf295_1*	Ferredoxin, leaf L-A				−1.1	
*POR1681..scf209_3*	Ferredoxin-NADP reductase (FNR)		1.1	1.5	−1.1	−1.5
*POR7078..scf295_1*	ATP synthase, gamma chain		1.4	1.7	−1.1	−1.4

**Table 4:** Differential expression of KEGG Orthology (KO)-annotated genes identified in pairwise comparisons between NR, NL (1-2), and LND conditions (extract from Table S3.2), expressed in log2 fold change (FC). ALG5, dolichyl phosphate β-glucosyltransferase; RPN2, dolichyldiphospho-oligosaccharide-protein subunit; OSTC, oligosaccharyltransferase complex subunit; and genes involved in polymerization, branching, and modification of polysaccharides. Differential expression color gradient was adjusted to max and min values of the overall table (blue: downregulated; red: upregulated).

		Fold Change (FC)				
Gene ID	Annotation (Abbreviation)	LND vs. NR	NL1 vs. NR	NL2 vs. NR	LND vs. NL1	LND vs. NL2
*POR6123..scf244_11*	dolichyl-phosphate β-glucosyltransferase (ALG5)	1.2				
*POR7179..scf289_17*	dolichyl-diphosphooligosaccharide-protein glycosyltransferase subunit (RPN2)		1.2		−1.1	
*POR5075..scf208_2*	oligosaccharyltransferase complex subunit (OSTC)	1.2	1.9	1.4		

## Data Availability

Data is contained within the article or Supplementary Materials. Raw transcriptomic data and processed files have been deposited in the NCBI Gene Expression Omnibus (GEO) under accession number GSE312736. Individual sample accessions range from GSM9352190 to GSM9352201.

## Supplementary Materials

The following supporting information can be downloaded at: https://www.mdpi.com/article/10.3390/md24010040/s1, Datasets: S1. Physiological characterization, kinetics, and statistical validation of *P. purpureum* cultivation; S2. Exopolysaccharide general (A) and monosaccharide (B) composition; S3. VST normalized gene expression of T1-T4 samples and corresponding gene annotations.; S4. Differential gene expression (DEGs) across experimental conditions; S5. Differential Regulation of GO Terms (GeneRatio, EF, ES) Across Pairwise Comparisons; S6. KEGG annotation summary and pathway enrichment distribution across pairwise comparisons; S7. Subcellular localization prediction of DEGs involved in nitrogen, sugar, and polysaccharide metabolism; S8. Carbohydrate-activated enzyme (CAZyme) gene annotation in the transcriptome; S9. CAZyme-coding DEG annotations and (sub)families distribution (%). Figures: S1. Volcano plots of DEGs across pairwise comparisons between all conditions; S2. K-term distribution among enriched KEGG categories (A) and KEGG Pathway distribution in ‘Metabolism’ category (B) S1. Tables: S1. Differentially Expressed Genes (DEGs) identified across nitrate conditions; S2. Differential gene expression and K-term annotations for nitrogen metabolism, photosynthesis, PPP, glycolysis, gluconeogenesis, oxidative phosphorylation; S3. Differential expression gene and K-term annotations for polysaccharide metabolism (CAZymes); S4. Differential gene expression of 33 CAZymes annotated in *P. purpureum*. All figures are in High Resolution. S5. CAZyme primer details used in the study for *P. purpureum*.
