# Supplementary material for: Transcriptomic Insights into Metabolic Reprogramming and Exopolysaccharide Synthesis in Porphyridium purpureum Under Gradual Nitrogen Deprivation
Source: Mar Drugs. 2026 Jan 13;24(1):40. doi: 10.3390/md24010040 (PMC12843361; doi:10.3390/md24010040)
Supplement: Supplementary file 1 [file marinedrugs-24-00040-s001.zip › Figure S1. Volcano plots of DEGs across pairwise comparisons between all conditions. .pdf]

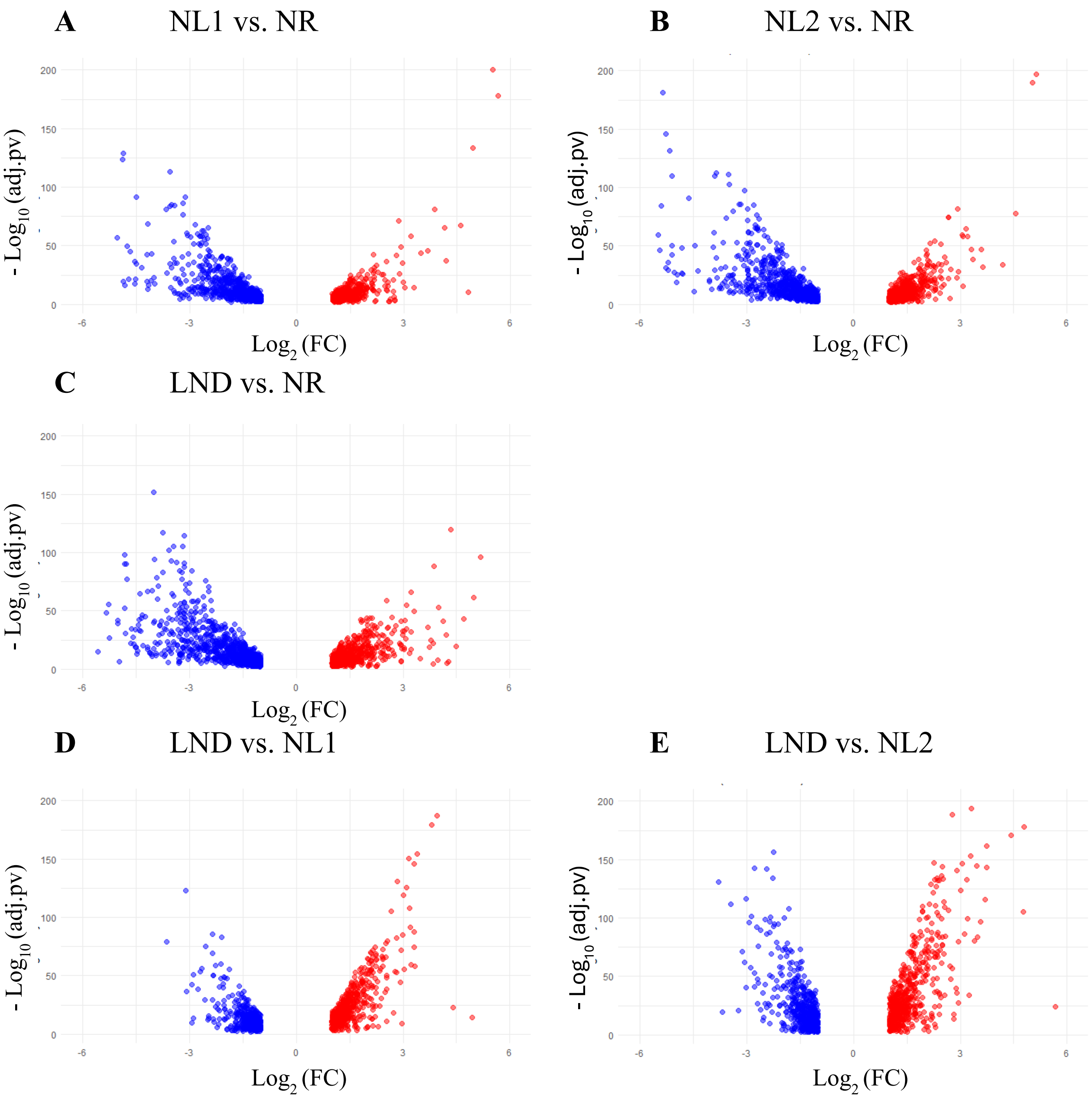

Figure S1. Complete set of volcano plots (A,B) of DEGs across pairwise comparisons (Figure 4 Complete). (A) NL1 vs. NR; (B) NL2 vs. NR, (C) LND vs. NR, (D) LND vs. NL1, (E) LND vs. NL2. Down- (blue) and up-regulated DEGs (red) are plotted according to their fold change (FC, log2) and their statistical significance (FDR, –log10(adj. p-value). FC threshold was set at $|\log2(FC)| \geq 1$ and FDR threshold was set at $-\log10(adj.pv) \geq 2$. Differential expression analysis was performed using DESeq2 on raw counts from all biological replicates, applying the likelihood ratio test (LRT) to identify DEGs. Volcano plots were generated from the resulting DESeq2 output.
