# Supplementary material for: Transcriptomic Insights into Metabolic Reprogramming and Exopolysaccharide Synthesis in Porphyridium purpureum Under Gradual Nitrogen Deprivation
Source: Mar Drugs. 2026 Jan 13;24(1):40. doi: 10.3390/md24010040 (PMC12843361; doi:10.3390/md24010040)
Supplement: Supplementary file 1 [file marinedrugs-24-00040-s001.zip › Figure S2. K-term distribution among enriched KEGG categories (A) and KEGG Pathway distribution in ‘Metabolism’ category (B) .pdf]

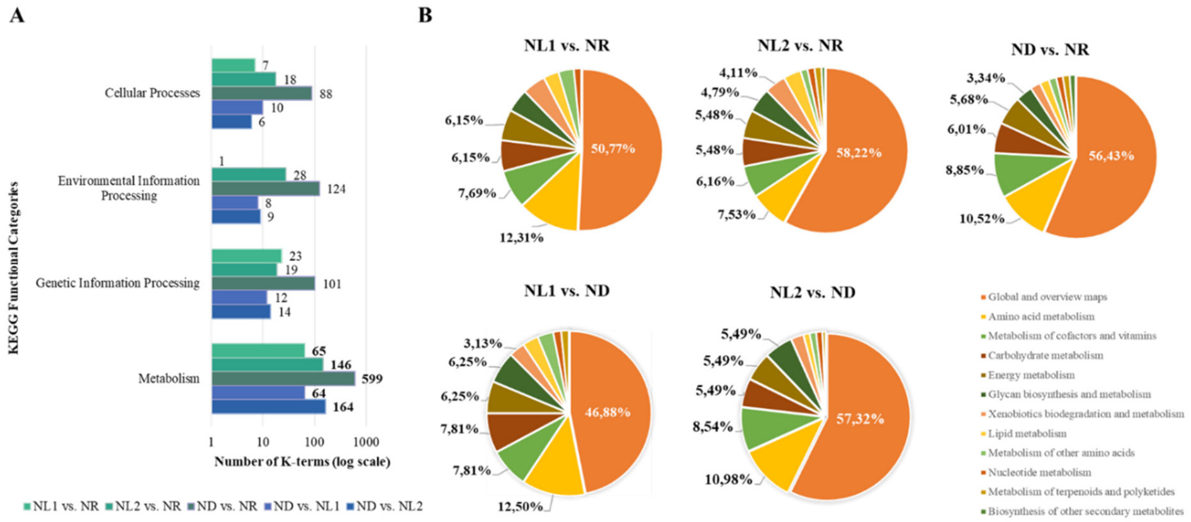

Figure S2. Distribution of K-terms among enriched KEGG functional categories (A) and KEGG Pathway distribution in 'Metabolism' category (B) across pairwise comparisons. Percentages in bold account for circa. 90 % of K-term annotations in each pairwise comparison.
