# Supplementary material for: Transcriptomic Insights into Metabolic Reprogramming and Exopolysaccharide Synthesis in Porphyridium purpureum Under Gradual Nitrogen Deprivation
Source: Mar Drugs. 2026 Jan 13;24(1):40. doi: 10.3390/md24010040 (PMC12843361; doi:10.3390/md24010040)
Supplement: Supplementary file 1 [file marinedrugs-24-00040-s001.zip › Table S2. Differential gene expression and K-term annotations.pdf]

Supporting information S2 Table description

Table S2.1 Differential gene expression and K-term annotations related to nitrogen metabolism in *P. purpureum*.

Table S2.2 Differential gene expression and K-term annotations related to photosynthesis.

Table S2.3 Differential gene expression and K-term annotations related to pentose phosphate pathway (PPP).

Table S2.4 Differential gene expression and K-term annotations related to glycolysis and gluconeogenesis.

Table S2.5 Differential gene expression and K-term annotations related to oxidative phosphorylation.

Table S2.1 Differential gene expression and K-term annotations related to nitrogen metabolism in *P. purpureum*. Differential expression of KEGG Orthology (KO)-annotated genes identified in pairwise comparisons between nitrogen-replete (NR), nitrogen-limited (NL1, NL2) and late-stage nitrogen-deprived (LND) conditions. “=” represents no significant differential expression. The annotated abbreviations correspond to those used to represent the genes in **Scheme 1**.

K-term	Gene description	Abbreviation	Gene ID	Consensus Subcellular Localization	NL vs. NR				LND vs. NR		LND vs. NL			
					NL1 vs. NR		NL2 vs. NR				LND vs. NL1		LND vs. NL2	
					Fold change (Log2FC)	-Log10 (FDR)	Fold change (Log2FC)	-Log10 (FDR)	Fold change (Log2FC)	-Log10 (FDR)	Fold change (Log2FC)	-Log10 (FDR)	Fold change (Log2FC)	-Log10 (FDR)
			POR6298..scf295_1	putative Lysosomal/vacuole transmembrane protein	-1.9	8.9	-1.7	9.6	=		2.9	44.2	2.7	59.0
K02575	High affinity nitrate transporter 2.5	NRT2.5	POR8948..scf295_1	putative Endoplasmic (reticulum) transmembrane protein	-1.8	11.4	-1.7	17.9	-4.4	43.5	-2.5	15.5	-2.6	21.5
			POR1427..scf295_1		-2.2	22.3	-2.4	27.6	-3.8	36.9	-1.5	6.4	-1.3	4.8
			POR7993..scf295_1		=		-1.4	4.6	=		=		=	
	Nitrate reductase NADH 2		POR6787..scf295_1	Cytosol	=		=		-2.7	23.9	-2.9	51.2	-2.8	53.0
K10534	Nitrate reductase NADH 1	NaR	POR8251..scf295_1	Cytosol	=		=		-2.9	29.4	-2.7	53.2	-2.7	44.4
	Nitrate reductase NADH 1		POR3077..scf295_1	Cytosol	=		=		-1.2	3.8	=		=	
K00262	Glutamate dehydrogenase	GluDH	POR3485..scf295_1	Cytosol	=		1.2	12.5	2.0	37.5	1.0	16.0	=	
K01915	Glutamine synthetase nodule isozyme	GlnS	POR7605..scf227_4	Chloroplastic. peripheral protein	=		1.2	14.0	=	=	-1.1	10.6	-1.6	36.0
K00366	Ferredoxin--nitrite reductase. chloroplastic	NiR	POR2445..scf236_6	Chloroplastic	=		=		-1.5	18.5	-2.3	58.6	-2.4	87.8

Table S2.2 Differential gene expression and K-term annotations related to photosynthesis. Differential expression of KEGG Orthology (KO)-annotated genes identified in pairwise comparisons between nitrogen-replete (NR), nitrogen-limited (NL1. NL2) and late-stage nitrogen-deprived (LND) conditions. “=” represents no significant differential expression.

K-term	Gene description	Abbreviation	Gene ID	Photosynthesis step	NL vs. NR				LND vs. NR		LND vs. NL			
					NL1 vs. NR		NL2 vs. NR				LND vs. NL1		LND vs. NL2	
					Fold change (Log2FC)	-Log10 (FDR)	Fold change (Log2FC)	-Log10 (FDR)	Fold change (Log2FC)	-Log10 (FDR)	Fold change (Log2FC)	-Log10 (FDR)	Fold change (Log2FC)	-Log10 (FDR)
K08902	Photosystem II lipoprotein Psb27	psb27	POR4156..scf229_5	PSII	2.0	25.4	2.2	52.5	=		=		-1.2	33.6
K02716	Oxygen-evolving enhancer protein 1. chloroplastic	psbO	POR0335..scf295_1	PSII-OEC	1.7	10.8	1.9	16.0	=		-1.1	10.8	-1.3	23.9
K02717	Photosystem II oxygen-evolving enhancer protein 2/ PsbP-like protein 1. chloroplastic	psbP	POR2049..scf244_11	PSII-OEC	=		1.3	9.7	=		-1.4	13.8	-1.7	26.0
K08901	Photosystem II oxygen-evolving enhancer protein 3 / hypothetical protein FVE85_6079	psbQ	POR3050..scf295_1	PSII-OEC	2.3	11.5	2.7	22.1	=		-1.5	14.3	-1.9	78.5
K02636	Cytochrome b6-f complex iron-sulfur subunit 1. cyanelle	petC	POR2665..scf244_11	cyt-b6	1.1	10.1	1.2	13.3	1.1	12.5	=		=	

Table S2.2 Differential gene expression and K-term annotations related to photosynthesis (continued).

K-term	Gene description	Abbreviation	Gene ID	Photosynthesis step	NL vs. NR				LND vs. NR		LND vs. NL			
					NL1 vs. NR		NL2 vs. NR				LND vs. NL1		LND vs. NL2	
					Fold change (Log2FC)	-Log10 (FDR)	Fold change (Log2FC)	-Log10 (FDR)	Fold change (Log2FC)	-Log10 (FDR)	Fold change (Log2FC)	-Log10 (FDR)	Fold change (Log2FC)	-Log10 (FDR)
K14332	Photosystem I subunit O	psaO	POR2912..scf227_4		2.5	12.0	2.4	9.9	=		-1.7	17.4	-1.5	4.0
K08907	Chlorophyll a-b binding protein 1B-21. chloroplastic	LHCA1	POR2835..scf295_9		2.0	14.4	2.2	26.0	1.0	7.2	=		-1.1	27.3
K08910	Chlorophyll a-b binding protein 1B-21. chloroplastic	LHCA4	POR6098..scf229_5	PSI	2.5	11.7	2.9	22.5	=		-1.5	4.8	-1.9	73.1
	Chlorophyll a-b binding protein. chloroplastic	LHCA4	POR9059..scf295_1		1.8	5.3	2.3	11.3	=		=		-1.4	50.4
	Chlorophyll a-b binding protein of LHCII type III. chloroplastic	LHCA4	POR2815..scf295_1		1.9	9.4	2.3	18.6	=		-1.1	9.2	-1.5	62.8
K02639	Ferredoxin. leaf L-A	petF	POR8201..scf295_1	FNR	=		=		=		-1.1	8.7	=	
K02641	Ferredoxin--NADP reductase. cyanelle	petH	POR1681..scf209_3	FNR	1.1	6.0	1.5	15.8	=		-1.1	12.0	-1.5	62.2
K02115	ATP synthase gamma chain. chloroplastic	ATPF1G. atpG	POR7078..scf295_1	ATP synthase chloroplastic	1.4	7.1	1.7	11.6	=		-1.1	13.7	-1.4	39.8

Table S2.3 Differential gene expression and K-term annotations related to pentose phosphate pathway (PPP). Differential expression of KEGG Orthology (KO)-annotated genes identified in pairwise comparisons between nitrogen-replete (NR), nitrogen-limited (NL1. NL2) and late-stage nitrogen-deprived (LND) conditions. "=" represents no significant differential expression. The annotated abbreviations correspond to those used to represent the genes in **Scheme 2**.

K-term	GenBank description	Abbreviation	Gene ID	Consensus Localization	NL vs. NR				LND vs. NR		LND vs. NL			
					NL1 vs. NR		NL2 vs. NR				LND vs. NL1		LND vs. NL2	
					Fold change (Log2FC)	-Log10 (FDR)	Fold change (Log2FC)	-Log10 (FDR)	Fold change (Log2FC)	-Log10 (FDR)	Fold change (Log2FC)	-Log10 (FDR)	Fold change (Log2FC)	-Log10 (FDR)
K01783	ribulose-phosphate 3-epimerase [EC:5.1.3.1]	RPE	POR7389..scf289_17	Chloroplastic	1.0	3.5	1.1	4.4	=		-1.1	8.5	-1.2	11.6
			POR0858..scf261_15	Chloroplastic	1.5	6.3	1.3	4.2	=		-1.4	5.7	-1.1	4.0
K00033	6-phosphogluconate dehydrogenase [EC:1.1.1.44 1.1.1.343]	PGD	POR4685..scf251_18	Cytosol	=		=		1.3	11.5	1.2	12.5	1.1	21.6
			POR4213..scf227_4	Ambiguous	=		=		-2.3	38.3	=		=	
K00036	glucose-6-phosphate 1-dehydrogenase [EC:1.1.1.49 1.1.1.363]	G6PD	POR9598..scf208_2	Cytosol	=		=		1.0	9.9	=		=	
K00948	ribose-phosphate pyrophosphokinase [EC:2.7.6.1]	PRPS	POR3179..scf295_1	Ambiguous	=		=		-1.4	5.5	-1.3	5.9	-1.2	5.3
K01057	6-phosphogluconolactonase [EC:3.1.1.31]	PGL	POR6162..scf295_1	Mitochondrial	=		=		1.1	8.5	=		=	

Table S2.4 Differential gene expression and K-term annotations related to glycolysis and gluconeogenesis. Differential expression of KEGG Orthology (KO)-annotated genes identified in pairwise comparisons between nitrogen-replete (NR), nitrogen-limited (NL1. NL2) and late-stage nitrogen-deprived (LND) conditions. "=" represents no significant differential expression. The annotated abbreviations correspond to those used to represent the genes in **Scheme 2**.

K-term	Gene description	Abbreviation	Gene ID	GenBank accession code	Consensus Localization	NL vs. NR				LND vs. NR		LND vs. NL			
						NL1 vs. NR		NL2 vs. NR				LND vs. NL1		LND vs. NL2	
						Fold change (Log2FC)	-Log10 (FDR)	Fold change (Log2FC)	-Log10 (FDR)	Fold change (Log2FC)	-Log10 (FDR)	Fold change (Log2FC)	-Log10 (FDR)	Fold change (Log2FC)	-Log10 (FDR)
K00845	glucokinase [EC:2.7.1.2]	GK	POR2873..scf295_1	KAA8499155.1	Ambiguous	=		=		1.3	17.7	1.1	17.1	=	
K01792	glucose-6-phosphate 1-epimerase [EC:5.1.3.15]	G6P 1-epi	POR4695..scf289_17	KAA8490972.1	Chloroplastic	1.2	5.0	1.2	5.7	=		=		=	
			POR2196..scf244_11	KAA8491994.1	Chloroplastic	1.7	15.8	1.6	15.5	1.6	17.2	=		=	
K03841	fructose-1.6-bisphosphatase I [EC:3.1.3.11]	FBP	POR7711..scf227_4	KAA8494850.1	Cytosol	1.2	4.1	1.7	15.9	=		=		-1.4	25.1
			POR3931..scf295_1	KAA8499964.1	Cytosol	1.6	9.7	2.0	25.5	=		=		-1.4	22.1
			POR8239..scf229_5	KAA8494021.1	Chloroplastic	=		=		1.2	17.1	=		=	
K01623	fructose-bisphosphate aldolase. class I [EC:4.1.2.13]	ALDO	POR3181..scf251_18	KAA8490818.1	Chloroplastic	1.6	15.3	1.6	13.1	=		=		=	
			POR9095..scf229_5	KAA8494018.1	Chloroplastic	1.3	7.0	1.7	18.3	1.2	8.2	=		=	
			POR1048..scf227_4	KAA8495104.1	Cytosol	-1.1	2.9	-1.5	4.6	-2.0	6.8	=		=	
K00927	phosphoglycerate kinase [EC:2.7.2.3]	PGK. pgk	POR3748..scf295_1	KAA8499853.1	Ambiguous	1.2	5.5	1.3	8.8	=		-1.2	10.5	-1.4	20.9
			POR4739..scf295_1	KAA8497794.1	Cytosol	=		=		1.6	23.9	1.3	18.7	1.1	34.9

Table S2.4 Differential gene expression and K-term annotations related to glycolysis and gluconeogenesis (continued).

K-term	Gene description	Abbreviation	Gene ID	Consensus Localization	NL vs. NR				LND vs. NR		LND vs. NL			
					NL1 vs. NR		NL2 vs. NR				LND vs. NL1		LND vs. NL2	
					Fold change (Log2FC)	-Log10 (FDR)	Fold change (Log2FC)	-Log10 (FDR)	Fold change (Log2FC)	-Log10 (FDR)	Fold change (Log2FC)	-Log10 (FDR)	Fold change (Log2FC)	-Log10 (FDR)
K15779	phosphogluco/pento-mutase [EC:5.4.2.2 5.4.2.7]	PGM2	POR0605..scf295_1	Cytosol	=		=		1.4	9.7	1.0	11.7	1.1	14.7
K01689	enolase 1/2/3 [EC:4.2.1.11]	ENO	POR6343..scf229_5	Cytosol	=		=		1.8	26.7	1.4	31.7	1.3	50.0
K00873	pyruvate kinase [EC:2.7.1.40]	PK	POR6038..scf208_2	Cytosol	=		=		1.2	18.6	=		=	
K01568	pyruvate decarboxylase [EC:4.1.1.1]	PDC	POR0764..scf295_9	Cytosol	1.4	4.0	1.4	4.8	3.8	24.6	2.4	72.5	2.4	132.2
K00382	dihydrolipoyl dehydrogenase [EC:1.8.1.4]	DLD	POR6810..scf209_3	Mitochondria	-1.3	7.1	-1.7	19.4	-2.0	20.4	=		=	
			POR3702..scf208_2	Mitochondria	=		=		-1.2	13.7	=		=	
			POR2889..scf236_6	Chloroplastic	=		=		=		-1.1	9.2	-1.5	26.0
K13953	alcohol dehydrogenase. propanol-preferring [EC:1.1.1.1]	ADH	POR6665..scf296_7	Cytosol	=		=		1.6	9.6	1.1	9.9	1.4	3.4
			POR0637..scf295_1	Chloroplastic	=		=		1.2	5.3	=		=	

Table S2.5 Differential gene expression and K-term annotations related to oxidative phosphorylation.

K-term	Gene description	Abbreviation	Gene ID	NL vs. NR				LND vs. NR		LND vs. NL			
				NL1 vs. NR		NL2 vs. NR				LND vs. NL1		LND vs. NL2	
				Fold change (Log2FC)	-Log10 (FDR)	Fold change (Log2FC)	-Log10 (FDR)	Fold change (Log2FC)	-Log10 (FDR)	Fold change (Log2FC)	-Log10 (FDR)	Fold change (Log2FC)	-Log10 (FDR)
K03942	(..) flavoprotein 1 [EC:7.1.1.2]	NADH (Complex I) components	POR0214..scf295_1	1.1	2.6	1.0	2.4	2.2	8.4	1.1	19.2	1.2	45.0
K03943	(..) flavoprotein 2 [EC:7.1.1.2]		POR6506..scf209_3	1.2	2.1	=		2.4	7.4	1.1	7.1	1.3	2.6
			POR6362..scf226_27	=	=	=	=	2.2	18.8	1.2	14.4	1.4	24.9
K03955	(..) 1 alpha/beta subcomplex 1. acyl-carrier protein		POR8552..scf209_3	1.5	9.3	1.6	9.0	1.3	6.8	=	=	=	=
			POR6026..scf295_1	1.4	18.2	1.4	14.8	=	=	=	=	=	=
K03963	(..)beta subcomplex subunit 7		POR6624..scf295_1	1.3	10.8	1.3	10.7	1.2	9.7	=	=	=	=
K03965	(..)1 beta subcomplex subunit 9		POR5888..scf295_1	1.1	8.3	1.1	5.9	=	=	=	=	=	=
K00416	(..) subunit 6	Ubiquinol-Cytochrome c Reductase (Complex III) components	POR4579..scf295_1	1.5	6.5	1.7	8.7	=	=	=	=	=	=
K00417	(..) subunit 7		POR0022..scf209_3	1.1	5.2	=	=	=	=	=	=	=	=
K02134	F-type H+-transporting ATPase subunit delta	F-ATPase. (Complex V) component	POR8536..scf227_4	1.0	4.8	=	=	=	=	=	=	=	=

Table S2.5 Differential gene expression and K-term annotations related to oxidative phosphorylation (continued).

K-term	Gene description	Abbreviation	Gene ID	NL vs. NR				LND vs. NR		LND vs. NL			
				NL1 vs. NR		NL2 vs. NR				LND vs. NL1		LND vs. NL2	
				Fold change (Log2FC)	Log10 (p-adj)	Fold change (Log2FC)	Log10 (p-adj)	Fold change (Log2FC)	Log10 (p-adj)	Fold change (Log2FC)	Log10 (p-adj)	Fold change (Log2FC)	Log10 (p-adj)
K02115	V-type H+-transporting ATPase 16kDa proteolipid subunit	V-type H^+-Transporting ATPase (V-ATPase)	POR6749..scf209_3	-1.1	7.5	-1.1	5.4	-1.1	5.0	=		=	
			POR7078..scf295_1	1.4	7.1	1.7	11.6	=		-1.1	13.7	-1.4	39.8
K02146	V-type H+-transporting ATPase subunit d		POR8327..scf227_4	=		1.2	13.8	=		=		=	
K02149	V-type H+-transporting ATPase subunit D		POR0161..scf295_9	=		=		1.1	5.2	=		=	
K02150	V-type H+-transporting ATPase subunit E		POR3560..scf209_3	1.3	6.3	1.3	6.5	1.0	4.8	=		=	
K02154	V-type H+-transporting ATPase subunit a		POR8744..scf229_5	=		=		1.0	3.1	1.0	3.1	=	
			POR2018..scf227_4	=		=		1.0	8.3	=		=	
			POR0353..scf227_4	=		=		-1.5	5.7	=		-1.3	7.6
K02155	V-type H+-transporting ATPase 16kDa proteolipid subunit		POR5789..scf209_3	1.5	11.6	1.0	2.4	=		-1.3	10.7	=	
