# Supplementary material for: Transcriptomic Insights into Metabolic Reprogramming and Exopolysaccharide Synthesis in Porphyridium purpureum Under Gradual Nitrogen Deprivation
Source: Mar Drugs. 2026 Jan 13;24(1):40. doi: 10.3390/md24010040 (PMC12843361; doi:10.3390/md24010040)
Supplement: Supplementary file 1 [file marinedrugs-24-00040-s001.zip › Table S3. Differential expression gene and K-term annotations for polysaccharide metabolism.pdf]

Supporting Information S3 Table description

S3 Table S1. Differential expression gene and K-term annotations for exopolysaccharide (EPS) precursors and nucleotide-sugar synthesis

S3 Table S2. Differential expression gene and K-term annotations for polymerization. branching and modification of polysaccharides

S3 Table S3. Differential expression gene and K-term annotations for carbohydrate-activated enzymes (CAZymes).

S3 Table S1. Differential expression gene and K-term annotations for exopolysaccharide (EPS) precursors and nucleotide-sugar synthesis.

K-term	Gene description	Abbreviation	Gene ID	Consensus Localization	NL vs. NR				LND vs. NR		LND vs. NL			
					NL1 vs. NR		NL2 vs. NR				LND vs. NL1		LND vs. NL2	
					Fold change (Log2FC)	-Log10 (FDR)	Fold change (Log2FC)	-Log10 (FDR)	Fold change (Log2FC)	-Log10 (FDR)	Fold change (Log2FC)	-Log10 (FDR)	Fold change (Log2FC)	-Log10 (FDR)
K15779	phosphoglucomutase / phosphopentomutase [EC:5.4.2.2 5.4.2.7]	PGM2	POR0605..scf295_1	Cytoplasmic	=		=		1.4	9.7	1.0	11.7	1.1	14.7
K17497	D-mannose 6-phosphate 1.6-phosphomutase	PMM	POR7125..scf295_1	Cytoplasmic	1.6	19.9	1.7	24.1	=		=		-1.1	21.9
K00012	UDP-glucose:NAD+ 6-oxidoreductase	UGDH	POR2992..scf295_1	Cytoplasmic	1.8	8.3	2.3	13.2	=		-1.9	55.6	-2.3	100.7
K00963	UTP:alpha-D-glucose-1-phosphate uridylyltransferase	UGP2. galU. galF	POR1962..scf209_3	Cytoplasmic	1.1	6.2	1.3	10.8	=		=		=	
K00621	glucosamine-phosphate N-acetyltransferase [EC:2.3.1.4]	GNPNAT1. GNA1	POR4056..scf295_1	Cytoplasmic	1.1	3.3	=		=		-1.1	3.8	=	
K00966	mannose-1-phosphate guanylyltransferase [EC:2.7.7.13]	GMPP	POR2740..scf296_7	Cytoplasmic	1.5	8.9	1.6	11.9	=		=		-1.0	15.9
K10046	GDP-D-mannose 3'. 5'-epimerase [EC:5.1.3.18 5.1.3.-]	GME	POR1248..scf295_1	Cytoplasmic	"="		1.1	8.4	=		=		=	
K08678	UDP-D-glucuronate carboxy-lyase (UDP-D-xylose-forming)	UXS1. uxs	POR4905..scf229_5	Cytoplasmic	"="		1.3	19.9	=		=		-1.2	36.0
K03331	xylitol:NADP+ 4-oxidoreductase (L-xylulose-forming)	DCXR	POR0983..scf227_4	Ambiguous	1.1	5.0	=		=		-1.2	6.5	=	

S3 Table S2. Differential expression gene and K-term annotations for polymerization. branching and modification of polysaccharides.

K-term	Gene description	Abbreviation	Gene ID	Consensus Localization	NL vs. NR				LND vs. NR		LND vs. NL			
					NL1 vs. NR		NL2 vs. NR				LND vs. NL1		LND vs. NL2	
					Fold change (Log2FC)	-Log10 (FDR)	Fold change (Log2FC)	-Log10 (FDR)	Fold change (Log2FC)	-Log10 (FDR)	Fold change (Log2FC)	-Log10 (FDR)	Fold change (Log2FC)	-Log10 (FDR)
K26501	oligosaccharyltransferase complex subunit OSTC	OSTC	POR5075..scf208_2	Ambiguous (ER transmembrane)	1.9	7.2	1.4	3.6	1.2	2.9	=		=	
K00729	dolichyl-phosphate beta-glucosyltransferase [EC:2.4.1.117]	ALG5	POR6123..scf244_11	Mitochondrial	=		=		1.2	3.7	=		=	
K12667	dolichyl-diphosphooligosaccharide--protein glycosyltransferase subunit 2 (ribophorin II)	RPN2	POR7179..scf289_17	Ambiguous (ER transmembrane)	1.2	4.2	=		=		-1.1	5.1	=	
K00688	glycogen phosphorylase [EC:2.4.1.1]	PYG	POR6603..scf209_3	Ambiguous (soluble)	=		=		1.3	21.6	=		=	
K00705	4-alpha-glucanotransferase [EC:2.4.1.25]	malQ	POR0525..scf295_1	Cytoplasmic	=		=		1.2	18.9	=		=	

S3 Table S3. Differential expression gene and K-term annotations for carbohydrate-activated enzymes (CAZymes).

K-term	Gene description	Abbreviation	Gene ID	NL vs. NR				LND vs. NR		LND vs. NL			
				NL1 vs. NR		NL2 vs. NR				LND vs. NL1		LND vs. NL2	
				Fold change (Log2FC)	-Log10 (FDR)	Fold change (Log2FC)	-Log10 (FDR)	Fold change (Log2FC)	-Log10 (FDR)	Fold change (Log2FC)	-Log10 (FDR)	Fold change (Log2FC)	-Log10 (FDR)
**K16055**	Alpha.alpha-trehalose-phosphate synthase UDP-forming A	GT20 (TPS)	POR6823..scf209_3	=		=		1.24	7.46	1.67	34.72	1.73	78.59
**K13676**	Glucoside xylosyltransferase 2	GT8 GXYLT	POR4276..scf295_1	1.02	5.56	1.33	9.51	=		-1.29	16.86	-1.63	29.77
	Glucoside xylosyltransferase 2	GT8 GXYLT	POR5778..scf295_1	=		=		-1.07	6.27	-1.60	19.53	-2.09	57.92
**K00748**	lipid-A-disaccharide synthase [EC:2.4.1.182]	GT19 (lpX)	POR1913..scf208_2	-2.26	28.85	-2.53	37.92	-3.07	39.40	=		=	
**K09480**	Digalactosyldiacylglycerol synthase 2. chloroplastic [Porphyridium purpureum]	GT4 (DGD)	POR8498..scf209_3	=		=		-1.38	7.19			-1.78	21.71
**K13679**	Granule-bound starch synthase 1. chloroplastic/amyloplastic [Porphyridium purpureum]	GH13 (WAXY)	POR3626..scf295_1	1.85	10.62	2.10	18.56	=		=		-1.24	29.28
**K07407/K03715**	Alpha-galactosidase	GH36 (galA/MGD)	POR5976..scf209_3	=		=		1.21	3.57	1.61	41.90	1.57	46.62
