# Supplementary material for: Transcriptomic Insights into Metabolic Reprogramming and Exopolysaccharide Synthesis in Porphyridium purpureum Under Gradual Nitrogen Deprivation
Source: Mar Drugs. 2026 Jan 13;24(1):40. doi: 10.3390/md24010040 (PMC12843361; doi:10.3390/md24010040)
Supplement: Supplementary file 1 [file marinedrugs-24-00040-s001.zip › Table S4. Differential gene expression of 33 CAZymes annotated in P.purpureum .pdf]

Table S4. Differential gene expression of 33 CAZymes annotated in *P.purpureum* according to Bhattacharya et al. (2013). Expression was normalized to the ARNr16S housekeeping gene. Relative quantification and fold changes (FC) were calculated using the $2^{-\Delta\Delta Ct}$ method (Rao et al., 2013). Asterisks indicate statistically significant differences from control conditions (grey cases, non-significative;*, $p < 0.05$; **$p<0.01$ calculated using Kruskal & Wallis and Wilcoxon statistical tests).

					NL vs. NR		LND vs. NR		LND vs. NL	
**Contig (study annotation)**	**Contig (Bhattacharya et al.)**	**Gene description**	**Cazyme family**	**Gene code ("GX")**	**FC**	**statistical significance**	**FC**	**statistical significance**	**FC**	**statistical significance**
POR8262..scf209_3	Contig_2500.2	β-glycosyltransferase	GT2	1	5.84	*	-1.22		-7.45	**
POR5523..scf295_1	Contig_3446.7	β-glycosyltransferase	GT2	2	2.56		-1.34		-3.48	**
POR0521..scf246_12	Contig_2108.2	α-glycosyltransferase	GT4	3	2.93	*	-1.14		-3.52	*
POR0856..scf295_1	Contig_3551.5	α-glycosyltransferase	GT4	4	2.44		1.13		-2.20	
POR9558..scf209_3	Contig_448.18	β-glycosyltransferase/ D-inositol-3-phosphate glycosyltransferase	GT4(-B)	5	1.42		1.79	*	1.71	
POR5657..scf209_3	Contig_2050.13	α.α-trehalose-6-phosphate synthase	GT20	6	2.78	*	-1.13		-3.20	*
POR3489..scf209_3	Contig_2186.9	β-glycosyltransferase related to β-1.3-galactosyl-Oglycosyl-glycoprotein β-1.6-N acetylglucosaminyltransferase	GT14	7	-3.03	*	-2.33	*	1.28	
POR4449..scf291_13	Contig_3385.23	α-fucosyltransferase	GT10	8	2.57		-1.34		-3.49	**
POR1493..scf295_1	Contig_3521.7	α-mannosyltransferases	GT32	9	1.56		-1.32		-1.98	
POR6466..scf209_3	Contig_3569.7	digalactosyldiacylglycerol	GT4	10	-1.18		1.44		2.05	
POR0279..scf296_7	Contig_4416.1	β-xylosyltransferase	GT14	11	1.13		-2.72	**	-3.08	**
POR7266..scf295_1	Contig_4418.1	glycosyl transferase (UDP-sulfoquinovose)	GT4	12	-1.00		-1.27		1.19	
POR6478..scf227_4	Contig_4476.21	α -mannosyltransferases	GT32	13	-1.19		1.58		2.42	
POR3663..scf209_3	Contig_448.16	α -mannosyltransferases	GT32	14	1.01		-1.37		-1.08	
POR5884..scf295_1	Contig_514.2	b-xylosyltransferases (β-glucuronosyltransferase)	GT14	15	-1.41	*	-1.11		1.50	

Table S4. (continued).

Contig (study annotation)	Contig (Bhattacharya et al.)	Gene description	Cazyme family	Gene code ("GX")	NL vs. NR		LND vs. NR		LND vs. NL	
					FC	statistical significance	FC	statistical significance	FC	statistical significance
POR5884..scf295_1	Contig_514.2	b-xylosyltransferases (β-glucuronosyltransferase)	GT14	15	-1.41	*	-1.11		1.50	
POR4547..scf295_1	Contig_597.4	β-glycosyltransferase distantly related to β-1.4-N-acetylgalactosaminyltransferase	GT25	16	-1.83		-1.69	*	1.32	
POR8187..scf295_1/POR7139..scf209_3	Contig_603.1	protein	GT41	17	-1.34		-1.36		1.57	
POR7290..scf218_34/POR7996..scf208_2	Contig_2179.7	β-glycosyltransferase	b-glycosyltransferase	18	2.30		-1.26		-2.69	
POR6738..scf209_3	Contig_2035.17	α-glycosyltransferase	GT4(-A)	19	1.20		-2.09	*	-2.57	*
POR4888..scf295_1	Contig_3478.15	β-glycosyltransferase	GT7	20	-1.16		-2.41	**	-2.08	*
POR6722..scf209_3	Contig_2035.10	β-xylosyltransferase	GT90	21	1.28		-3.20		-3.20	*
POR7326..scf295_1	Contig_2293.15	monogalactosyldiacylglycerol synthase	GT28	22	1.79	*	-2.05	*	-3.71	**
POR8498..scf209_3	Contig_496.8	digalactosyldiacylglycerol synthase	GT4(-C)	23	-3.31		-6.98	**	-1.49	
POR8522..scf208_2	Contig_636.1	α -mannosyltransferases	GT32	24	1.05	**	-1.05		-1.03	
POR6353..scf208_2	Ppu.Contig_2053.9	β-glycosyltransferase distantly related to N-acetylglucosaminyl-proteoglycan β-1.4-glucuronosyltransferases/ exostocin-1c	GT47	25	5.25		1.04		-4.80	**
POR5778..scf295_1	Ppu.contig_2062.25	NDP-sugar α-glycosyltransferase distantly related to α-xylosyltransferase	GT8(-B)	26	-1.17		-2.19	*	-1.81	
POR2297..scf295_1	Ppu.contig_2088.2	α-glycosyltransferase/xylosyltransferase	GT8	27	2.01		-2.17		-4.64	***
POR2060..scf227_4	Ppu.contig_2111.9	a-glycosyltransferase/β-arabinofuranosyltransferase	GT77	28	-1.27		1.58		2.77	

					NL vs. NR		LND vs. NR		LND vs. NL	
**Contig (study annotation)**	**Contig (Bhattacharya et al.)**	**Gene description**	**Cazyme family**	**Gene code ("GX")**	**FC**	**statistical significance**	**FC**	**statistical significance**	**FC**	**statistical significance**
POR1088..scf222_8	Ppu.contig_3435.11	b-glycosyltransferase/β-1.4-N-acetylglucosaminyltransferase	GT61	29	-1.49		-1.39		1.23	
POR5159..scf295_1	Ppu.contig_3446.8	NDP-sugar	GT8	30	-1.79	*	-1.09		1.99	
POR4276..scf295_1	Ppu.contig_3446.9	NDP-sugar α-glycosyltransferase distantly related to α-xylosyltransferase	GT8(-A)	31	-1.95		-1.49		1.60	
	Ppu.contig_3473.1	NDP-sugar	GT8(-A)	32	-1.67		-1.24		2.22	
POR3691..scf244_11	Ppu.contig_3623.1	α-glycosyltransferase distantly related to arabinosyltransferase	GT77	33	-1.72		-3.33	**	-1.60	
