# Supplementary material for: Transcriptomic Insights into Metabolic Reprogramming and Exopolysaccharide Synthesis in Porphyridium purpureum Under Gradual Nitrogen Deprivation
Source: Mar Drugs. 2026 Jan 13;24(1):40. doi: 10.3390/md24010040 (PMC12843361; doi:10.3390/md24010040)
Supplement: Supplementary file 1 [file marinedrugs-24-00040-s001.zip › Table S5 CAZyme primer details used in the study for P.purpureum.pdf]

Table S5. Primer description of CAZymes used in the study for *P. purpureum*.

Contig (study annotation)	Contig (Bhattacharya et al.)	Cazyme family	Gene code ("GX")	Forward primer (5′–3′)	Tm (°C)	Reverse primer (5′–3′)	Tm (°C)	Amplicon size (bp)
POR8262..scf209_3	Contig_2500.2	GT2	1	ACGCGGTACTGAGTTGCAC	59.9	CTGGTCGCCATACGGTAAAC	60	284
POR5523..scf295_1	Contig_3446.7	GT2	2	AATCAAGAACACCGCCAAAG	60.1	TGGTTGTCTGATCCACGGTA	60	282
POR0521..scf246_12	Contig_2108.2	GT4	3	GACAGGTTTATGGCCGAAGA	60.1	CGCATCAACGACATTTCAAC	60.1	314
POR0856..scf295_1	Contig_3551.5	GT4	4	CATCTGTGTTGATGGCTTGG	60.1	ATGGTCTGGACCTTGTACGC	60	292
POR9558..scf209_3	Contig_448.18	GT4(-B)	5	TGGACGTGAAGAAGAACGTG	59.9	GCCATCACACTGAAGCTGAA	60	307
POR5657..scf209_3	Contig_2050.13	GT20	6	TGGTACGGGAGGACTAGGTG	60	TCCAAGTGATGAGCGATCTG	59.9	288
POR3489..scf209_3	Contig_2186.9	GT14	7	CTGAGCGCACTATACCACGA	60	CGCCGACAGGTAAATCAAAT	60	318
POR4449..scf291_13	Contig_3385.23	GT10	8	GTCCGAAGACATGTGTGGTG	60	ATTTTCCGTACGCGTCTGTC	60.1	316
POR1493..scf295_1	Contig_3521.7	GT32	9	CTATGCGGGTCTAACGTGGT	60	AAAATATCGGCCTTGTGCTG	60.1	284
POR6466..scf209_3	Contig_3569.7	GT4	10	ACAATGCGAGAAGCGAGTTT	60	CTCAGCCAAGGAACCAGAAG	60	314
POR0279..scf296_7	Contig_4416.1	GT14	11	GGTGTTCGGAGAAGAAGCTG	60	GGTTAAAGGCGACCATGCTA	60.1	281
POR7266..scf295_1	Contig_4418.1	GT4	12	AAGACGCTGCTCAAGTGGTT	60.1	TGCGTGTGGTACGAGAAAAG	59.9	296
POR6478..scf227_4	Contig_4476.21	GT32	13	GATCCAGCCAGACGAATGAT	60	ATCAACAGGAGCACCAAAGG	60.1	281
POR3663..scf209_3	Contig_448.16	GT32	14	CTTCTGCTGTTGCTGAGCTG	60.1	AGCTTGCGGAGTTCATCACT	60	301
POR5884..scf295_1	Contig_514.2	GT14	15	GTGCATTCGACTGAAGCAAA	60	ATGCGATCCATCAGGTTCTC	60	290
POR5884..scf295_1	Contig_514.2	GT25	16	ACCTGGAGTCATGTCCTTCG	60.1	CTTCAAGGTGGCCATCATCT	60.1	296
POR4547..scf295_1	Contig_597.4	GT41	17	GGTACCATAAGCCTGGCAAA	60	TTCACTTTCCACTCGCTCCT	60	282
POR8187..scf295_1/POR7139..scf209_3	Contig_603.1	b-glycosyltransferase	18	GATTTCGACACAGCGACAGA	60	GATGGACTTGAGGGAGGTGA	60	300

Table S5. (continued)

Contig (study annotation)	Contig (Bhattacharya et al.)	Cazyme family	Gene code ("GX")	Forward primer (5′–3′)	Tm (°C)	Reverse primer (5′–3′)	Tm (°C)	Amplicon size (bp)
POR7290..scf218_34/ POR7996..scf208_2	Contig_2179.7	GT4 (-A)	19	CATGGCTCAGGAGTTTGACA	59.8	ACAGGCCGTCGTATGGATAG	60	317
POR6738..scf209_3	Contig_2035.17	GT7	20	GGTGGCAGAGAGAGCGATAC	60	TACAGAAGCAGAGCGGGAAT	60	297
POR4888..scf295_1	Contig_3478.15	GT7	21	TCGGTTTCCAAGACAACTCC	60.1	CCTGGTGTTCTTCGACCATT	60	285
POR6722..scf209_3	Contig_2035.10	GT90	22	TCCACGGGTATCTTGACACA	60	TCCAGTTGGATCTTGTGCAG	59.8	320
POR7326..scf295_1	Contig_2293.15	GT28	23	CGGTGAGTGAAGCTGATGAA	60	CGACTCTGGCGGTGTATTTT	60.1	290
POR8498..scf209_3	Contig_496.8	GT4 (-C)	24	GGATTTGCAAACGAAATGCT	60.1	CCACGTGTCTCCCTTGAACT	60.2	300
POR8522..scf208_2	Contig_636.1	GT32	25	TACTGGGACGCTTTGCTATG	61	TCACCCTTCATCCTGTTTGG	61	307
POR6353..scf208_2	Ppu.Contig_2053.9	GT47	26	GCTTTGGAACGGCTGAAGTA	62	GCAGGGATGAAGCAGGTATTT	62	299
POR2297..scf295_1	Ppu.contig_2088.2	GT8	27	TGGCCTGAACTACCTGAATG	61	CGATTGCCGTGGAGAATAGA	61	294
POR2060..scf227_4	Ppu.contig_2111.9	GT77	28	AGCAGCTCACGAAGATGAAG	62	CCGCACATGACATCGTAGAA	62	310
POR1088..scf222_8	Ppu.contig_3435.11	GT61	29	AGGCACTTACGTGGAACAATAA	62	GTAAACCGCGACATTGGAAATC	62	322
POR5159..scf295_1	Ppu.contig_3446.8	GT8	30	GCTGGATCTGAAGACCAGAAA	61	GAGACATTGCACGAAGGATCTA	61	306
POR4276..scf295_1	Ppu.contig_3446.9	GT8(-A)	31	CACATGTCGACCGGTTACTATAC	62	GATTCAGCGCAAATCCGATTAAA	62	392
	Ppu.contig_3473.1	GT8(-A)	32	CTTGGATACGGATACGCTGATT	62	CTTGGTCCGCAAAGTAAATGTC	61	317
POR3691..scf244_11	Ppu.contig_3623.1	GT77	33	GCACTGCCATGAACTTTGTATG	62	ATCGAGCCACACCGTATCTA	62	302
